# Altered Gut Microbiota in Patients With Peutz–Jeghers Syndrome

**DOI:** 10.3389/fmicb.2022.881508

**Published:** 2022-07-13

**Authors:** Sui Wang, Gang Huang, Jue-Xin Wang, Lin Tian, Xiu-Li Zuo, Yan-Qing Li, Yan-Bo Yu

**Affiliations:** ^1^Department of Gastroenterology, Qilu Hospital of Shandong University, Jinan, China; ^2^Laboratory of Translational Gastroenterology, Qilu Hospital of Shandong University, Jinan, China

**Keywords:** Peutz-Jeghers syndrome, intestinal microflora, bacteria, fungal microbiota, dysbiosis

## Abstract

**Background:**

Peutz–Jeghers syndrome (PJS) is a rare genetic disorder characterized by the development of pigmented spots and gastrointestinal polyps and increased susceptibility to cancers. It remains unknown whether gut microbiota dysbiosis is linked to PJS.

**Aim:**

This study aimed to assess the structure and composition of the gut microbiota, including both bacteria and fungi, in patients with PJS and investigate the relationship between gut microbiota dysbiosis and PJS pathogenesis.

**Methods:**

The bacterial and fungal composition of the fecal microbiota was analyzed in 23 patients with PJS (cases), 17 first-degree asymptomatic relatives (ARs), and 24 healthy controls (HCs) using 16S (MiSeq) and ITS2 (pyrosequencing) sequencing for bacteria and fungi, respectively. Differential analyses of the intestinal flora were performed from the phylum to species level.

**Results:**

Alpha-diversity distributions of bacteria and fungi indicated that the abundance of both taxa differed between PJS cases and controls. However, while the diversity and composition of fecal bacteria in PJS cases were significantly different from those in ARs and HCs, fungal flora was more stable. High-throughput sequencing confirmed the special characteristics and biodiversity of the fecal bacterial and fungal microflora in patients with PJS. They had lower bacterial biodiversity than controls, with a higher frequency of the Proteobacteria phylum, Enterobacteriaceae family, and *Escherichia-Shigella* genus, and a lower frequency of the Firmicutes phylum and the Lachnospiraceae and Ruminococcaceae families. Of fungi, *Candida* was significantly higher in PJS cases than in controls.

**Conclusion:**

The findings reported here confirm gut microbiota dysbiosis in patients with PJS. This is the first report on the bacterial and fungal microbiota profile of subjects with PJS, which may be meaningful to provide a structural basis for further research on intestinal microecology in PJS.

## Introduction

Peutz–Jeghers syndrome (PJS) is an autosomal-dominant hereditary disease characterized by gastrointestinal hamartomatous polyps, mucocutaneous melanin deposits, and a predisposition for both gastrointestinal and non-gastrointestinal cancer (Giardiello et al., 1987; Sengupta and Bose, 2019; Tacheci et al., 2021). Colorectal, gastric, small intestinal, breast, and pancreatic cancers are the most common malignancies associated with PJS (Klimkowski et al., 2021; Sandru et al., 2021; Tacheci et al., 2021). The primary manifestation of PJS is the presence of hamartoma polyps throughout the gastrointestinal tract. Germline mutations of the tumor suppressor gene, *LKB1/STK11*, are the cause of PJS (Daniell et al., 2018; Altamish et al., 2020; Zhang et al., 2020). However, the function and activity of *LKB1*, which encodes for liver kinase B1 (LKB1), are still being researched. LKB1 plays an important role in cellular metabolism, cell polarity, DNA damage repair, and epithelial–mesenchymal transformation. STK11 inhibits the mammalian target of rapamycin (mTOR) pathway (Martin and St Johnston, 2003; Baas et al., 2004; Zhang et al., 2021), and its mutations are linked to various tumor types in both syndromic and sporadic contexts (Carretero et al., 2010).

Inflammation appears to play an important role in PJS pathogenesis. LKB1 regulates inflammation and particular inflammatory cytokines, including cyclooxygenase (COX)-2, interleukin (IL)-6, and IL-11, and stimulates intestinal polyposis and gastrointestinal tumorigenesis in patients with PJS and animal models (De Leng et al., 2003; Udd et al., 2004; Ollila et al., 2018; Poffenberger et al., 2018). STK11-AMPK-mTOR signaling also regulates the occurrence and development of PJS. While oral selective mTOR inhibitors, such as rapamycin and everolimus, have been successfully used (van Lier et al., 2010; Klümpen et al., 2011), the feasibility of using these agents for chemoprevention remains unknown given the small sample sizes of prior studies and their high cost and toxicity (de Brabander et al., 2018). While mutations in *STK11* are the primary cause of PJS, they are not present in all patients, suggesting that there is some genetic heterogeneity. Indeed, mutations in other genes, including *FHIT* (Zhao et al., 2003), *MMR* (Zhang et al., 2020), and *SLX4* (Gu et al., 2021), are also associated with PJS. There is some controversy in the literature about the involvement of these and other genes, including *STK11* variants, in PJS as a result of different screening technologies and small sample sizes (Buchet-Poyau et al., 2002; Daniell et al., 2018). More robust studies are needed to define how current mutations impact the development of PJS and identify additional variants associated with this syndrome.

Multiple benign gastrointestinal hamartomatous polyps associated with PJS can cause local bleeding, occlusion, and intussusception, and increase the risk of cancer (Sandru et al., 2021). While diagnostics, such as advanced endoscopy, and surveillance regimens help identify PJS at an early stage, acute and chronic complications affect patients' quality of life and decrease life expectancy.

Recent technical developments in sequencing have made it possible to investigate the correlations between microbiota dysbiosis and disease. The microbiota plays an important role in many digestive diseases, including inflammatory bowel disease (IBD) (Xu et al., 2018; Beheshti-Maal et al., 2021), colorectal adenoma (CRA) (Feng et al., 2015), irritable bowel syndrome (IBS) (Gu et al., 2019; Takakura and Pimentel, 2020), and colorectal carcinoma (CRC) (Janney et al., 2020; Qin et al., 2021). Each individual has a unique composition of intestinal bacteria that are influenced by several factors, such as lifestyle, diet, geography, environment, genetics, and medications (Falony et al., 2016; Lynch and Pedersen, 2016; Deschasaux et al., 2018; He et al., 2018). The human gut is also a habitat for numerous fungi, collectively known as the mycobiome (Enaud et al., 2018; Chin et al., 2020), the configuration of which is influenced by geography, ethnicity, diet, and urbanization, and can vary significantly across populations (Lloyd-Price et al., 2016; Davenport et al., 2017; Lkhagva et al., 2021). The make-up of the intestinal microbiota, combined with population characteristics, such as lifestyle, ethnicity, geographical region, and diet, affects the health and metabolism of the host (Sun et al., 2021). Host metabolism is also regulated by certain microbial species, and particular microorganism types in the bowel are involved in exacerbating tumor growth (Deschasaux et al., 2018).

The structure and function of the microbiota in patients with PJS and how they differ from the microbiota of healthy individuals remain unknown. It is likely that the altered intestinal environment, often including massive or scattered intestinal polyps and an elevated frequency of malignancies, may affect the intestinal flora of individuals with PJS. This study analyzed the components of the microbiota, including bacteria and fungi, in patients with PJS to identify potential microecological biomarkers of this disease. While the cohort was limited in size, this is the first comparison of the bacterial and fungal flora of patients with PJS and their healthy relatives.

## Methods

### Study Population

All recruited patients with PJS were inpatients at Qilu Hospital of Shandong University, China. Individuals with (1) a positive family history of PJS and any number of histologically confirmed PJS polyps or characteristic, prominent, mucocutaneous pigmentation, or (2) a negative family history of PJS and ≥3 histologically confirmed PJS polyps or any number of histologically confirmed PJS polyps and characteristic pigmentation were confirmed as having PJS (Dhroove, 2017).

Individuals were excluded if they had (1) received pharmacological agents, such as antidepressants, antidiarrheal agents, laxatives, and antibiotics, or probiotic supplements 4 weeks prior to the study, (2) had an enterectomy, an acute gastrointestinal hemorrhage, or intestinal neoplasia, or (3) were considered unsuitable for an enteroscopy as a result of coagulopathy, severe cardiopulmonary diseases, or pregnancy.

**Figure 1:**
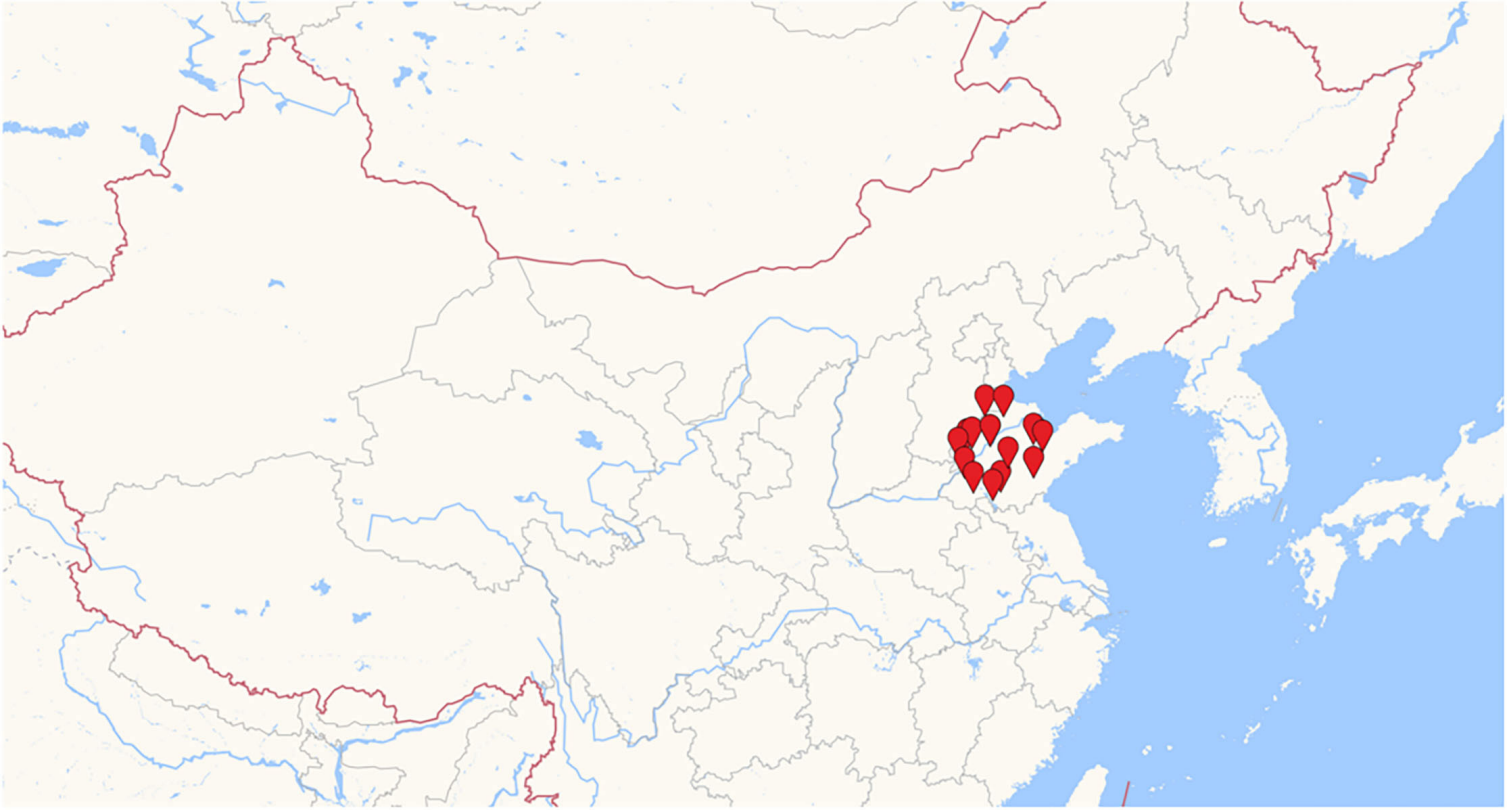
Geographical distribution of the studied populations. Only individuals of Han Chinese ethnicity living in Shandong and the neighboring province were included, mitigating the effects of ethnic, dietary, and geographic differences on the results.

A total of 23 patients with PJS, 17 asymptomatic relatives (ARs), and 24 healthy controls (HCs) were involved in this study. The participants were from 15 families in the Shandong Province in China (Figure 1), and all were Chinese Han. Notably, 23 patients with PJS had a median age of 28.61 years (range: 3–56 years), and 15 of them were men (65.2%); 17 first-degree healthy relatives had a median age of 33.76 years (range: 1–57 years), and six of them were men (35.2%).

A total of 24 healthy volunteers were examined to ensure that they had no clinical symptoms, a history of gastroenterology disorders, histological or endoscopic signs of tumor or IBD, and selected as the control group. They had a median age of 26.75 years (range: 22–54 years), and 15 of them were men (62.5%). The HCs included seven spouses of the patients with PJS and 17 volunteers who were recruited from Shandong University.

The families were interviewed and sampled in the hospital or their homes, and family members did not necessarily live in the same household. Detailed information about the participants is included in Table 1.

**Table 1:** Demographic and clinical data of patients with Peutz–Jeghers syndrome (PJS), asymptomatic relatives (ARs), and healthy controls (HCs).

		**PJS Patients**	**ARs**	**HCs**
Number		23	17	24
Gender (%female)		15:8 (34.78%)	6:11 (64.70%)	15.9 (37.50%)
Age, mean (min–max)		28.61 (3 ~ 56)	33.76 (1 ~ 57)	26.75 (22 ~ 54)
BMI, mean (min–max)		22.17 (14.88 ~ 33.2)	23.46 (14.79 ~ 31.64)	23.61 (17.78–34.89)
Disease duration, median (min–max)		16 (1 ~ 42)	NA	NA
Previous abdominal surgery		16 (69.57%)	0	0
Family history		14 (60.87%)	3	NA
Sporadic PJS		9 (39.13%)	NA	NA
Malignancy		3 (13.04%)	0	0
Mucocutaneous pigmentation		23 (100%)	0	NA
Distribution of Polyps, *n* (%)	Stomach-Colon	7 (30.43%)	NA	NA
	Small intestine-colon	9 (39.13%)	NA	NA
	Small intestine	2 (8.70%)	NA	NA
	Small intestine-rectum	1 (4.35%)	NA	NA
	No polypectomy	4 (17.39%)	NA	NA

*The mean and median values are given, with the minimum and maximum values in parentheses. NA, not applicable; BMI, body mass index*.

This study was approved by the medical Ethics Committee at Qilu Hospital of Shandong University, and procedures were conducted according to the 1975 Declaration of Helsinki. Prior to their participation, all volunteers provided informed written consent.

### Fecal Sample Collection, DNA Extraction, and Pyrosequencing

Fecal samples were obtained from 69 participants, and all samples were put on ice without preservatives, transported to the laboratory, and stored at −80°C within 2 h of collection. The samples were shipped to Major-Bio (Shanghai, China) for high-throughput sequencing. The E.Z.N.A.® soil DNA Kit (Omega Bio-Tek, Norcross, GA, USA) was used to extract microbial DNA from 69 samples according to the protocol of the manufacturer. The final DNA concentration and purification were determined using a NanoDrop 2000 UV-vis spectrophotometer (Thermo Scientific, Wilmington, USA), and the DNA quality was determined using 1% agarose gel electrophoresis. Polymerase chain reaction (PCR) (ABI Gene Amp 9700, ABI, USA) was used to amplify the V3–V4 region of the bacterial 16S rRNA gene using primers 338F (ACTCCT ACGGGAGGCAGCAG) and 806R (ACTCCTACGGGA GGCAGCAG) and the Trans Start Fast pfu DNA polymerase (Trans Gen, Beijing, China). For fungi, primers ITS1F (5′-CTTGGTCATTTAGAGGAAGTAA-3′) and ITS2R (5′-GCTGCGTTCTTCATCGATGC-3′) were used to amplify the ITS1 region. The resulting PCR products were extracted from a 2% agarose gel, further purified using the AxyPrep DNA Gel Extraction Kit (Axygen Biosciences, Union City, CA, USA) and quantified using QuantiFluor^™−^ST (Promega, USA).

### Illumina MiSeq Sequencing and Processing of the Floral Data

Purified amplicons were pooled in equimolar amounts and paired-end sequenced (2 × 300) on an Illumina MiSeq platform (San Diego, USA) in accordance with standard protocols from Bio-Pharm Technology Co. Ltd in Majorbio. Raw FastQ files were demultiplexed, quality-filtered (Trimmomatic), and merged (FLASH). The abundance of operational taxonomic units (OTUs) was normalized by a standard sequence number according to the least sequence of the samples and clustered with a 97% similarity cutoff (UPARSE, version 7.1 http://drive5.com/uparse). Chimeric sequences were identified and removed (UCHIME). The taxonomy was analyzed against the Silva (SSU132) 16S rRNA and Unite 8.0 fungi databases using a confidence threshold of 70%. Data analysis was performed on the open online platform of the Majorbio Cloud Platform. QIIME was used to determine the alpha- and beta-diversity and conduct a principal coordinate analysis (PCoA). The execution of linear discriminant analysis effect size (LEfSe) was accomplished using the LEfSe tool. The PRIMER 6 software package (PRIMER-E Ltd., Luton, UK) similarity test (ANOSIM) was used to measure differences in the fecal flora communities of the PJS, AR, and HC groups. To identify different functional groups within the bacterial and fungal communities and link their relative abundance, the PICRUSt 2 software and Kyoto Encyclopedia of Genes and Genomes (KEGG) database were used for bacteria, and a recently developed open annotation tool (FunGuild, Fungi Functional Guild) was used for fungi. These modes were further subdivided into specific guilds of fungi or bacteria that shared similar modes. Sequence data were deposited to the NCBI Sequence Read Archive under BioProject accession numbers PRJNA802067 and PRJNA838192.

### Statistical Analyses

Bacteria and fungi were assessed at all levels, phylum, class, order, family, genus, and species, and the data sequences were analyzed using several different scales. Categorical data were compared using a chi-square test. A *p* < 0.05 was defined as statistically significant. The SPSS statistical package (version 26.0) was used to analyze the data.

## Results

### Clinical Characteristics of the Study Cohort at Baseline

Clinical characteristics were compared between the controls and patients with PJS, and significant differences in age, sex, and body mass index (BMI) were observed (*p* > 0.05). The demographics and PJS characteristics of the subjects are shown in Table 1. Lifestyle and clinical data, including age, sex, ethnicity, geography, bowel habits, dietary habits, smoking and drinking status, and BMI, were collected for all 64 individuals to assess whether any factor had an excessive impact on the outcomes.

### Patients With PJS Have Significantly Lower Bacterial Diversity Than Healthy Individuals

**Figure 2:**
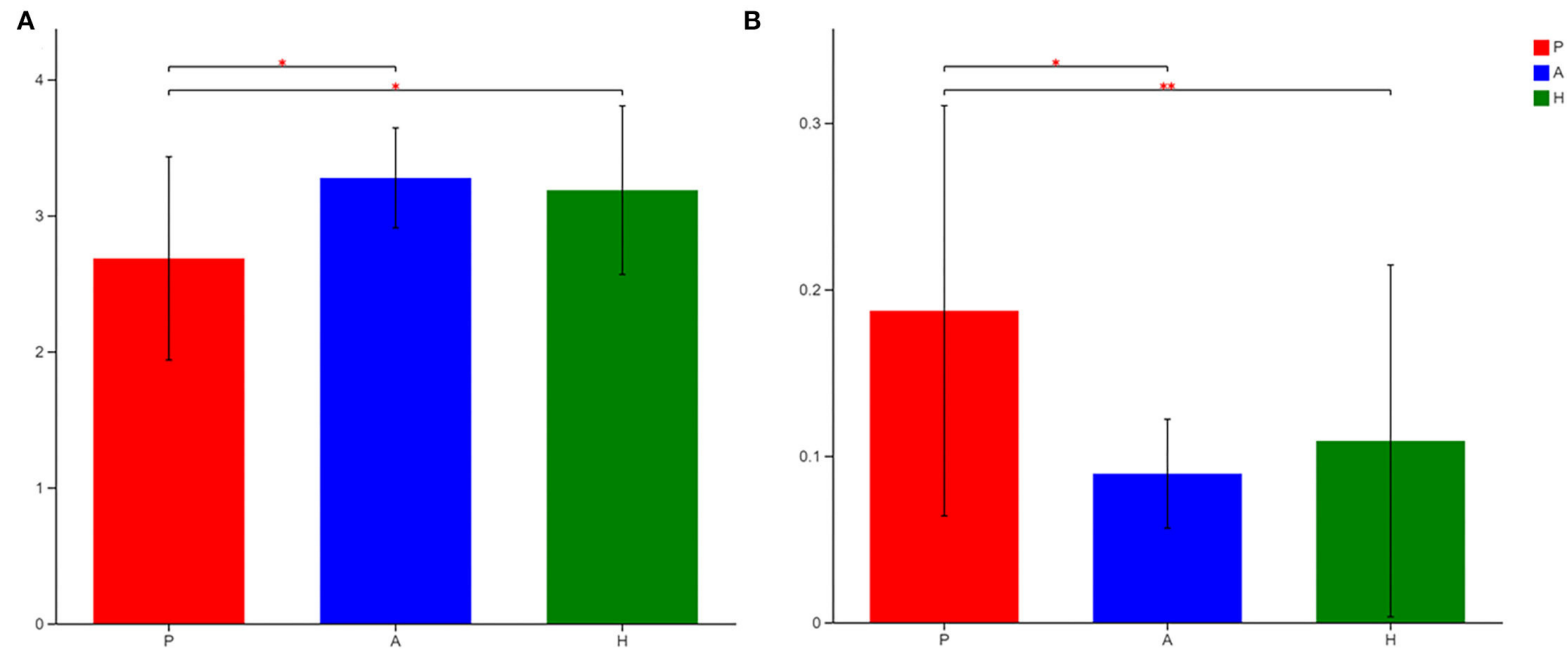
Alpha-diversity of the gut bacteria in different groups at the OTU level. **(A)** Differences in the Shannon diversity index among Peutz–Jeghers syndrome (PJS) patients (group P), asymptomatic relatives (group A), and healthy controls (group H). The Shannon index described the diversity of the bacterial microbiota at the OTU level (Wilcoxon rank–sum test, *p < 0.05). The higher the Simpson index value, the higher the bacterial biodiversity. **(B)** The Simpson index described the diversity of the bacterial microbiota at the operational taxonomic unit (OTU) level (Wilcoxon rank–sum test, *p < 0.05; **p < 0.01). The higher the Simpson index value, the lower the microbial biodiversity.

**Figure 3:**
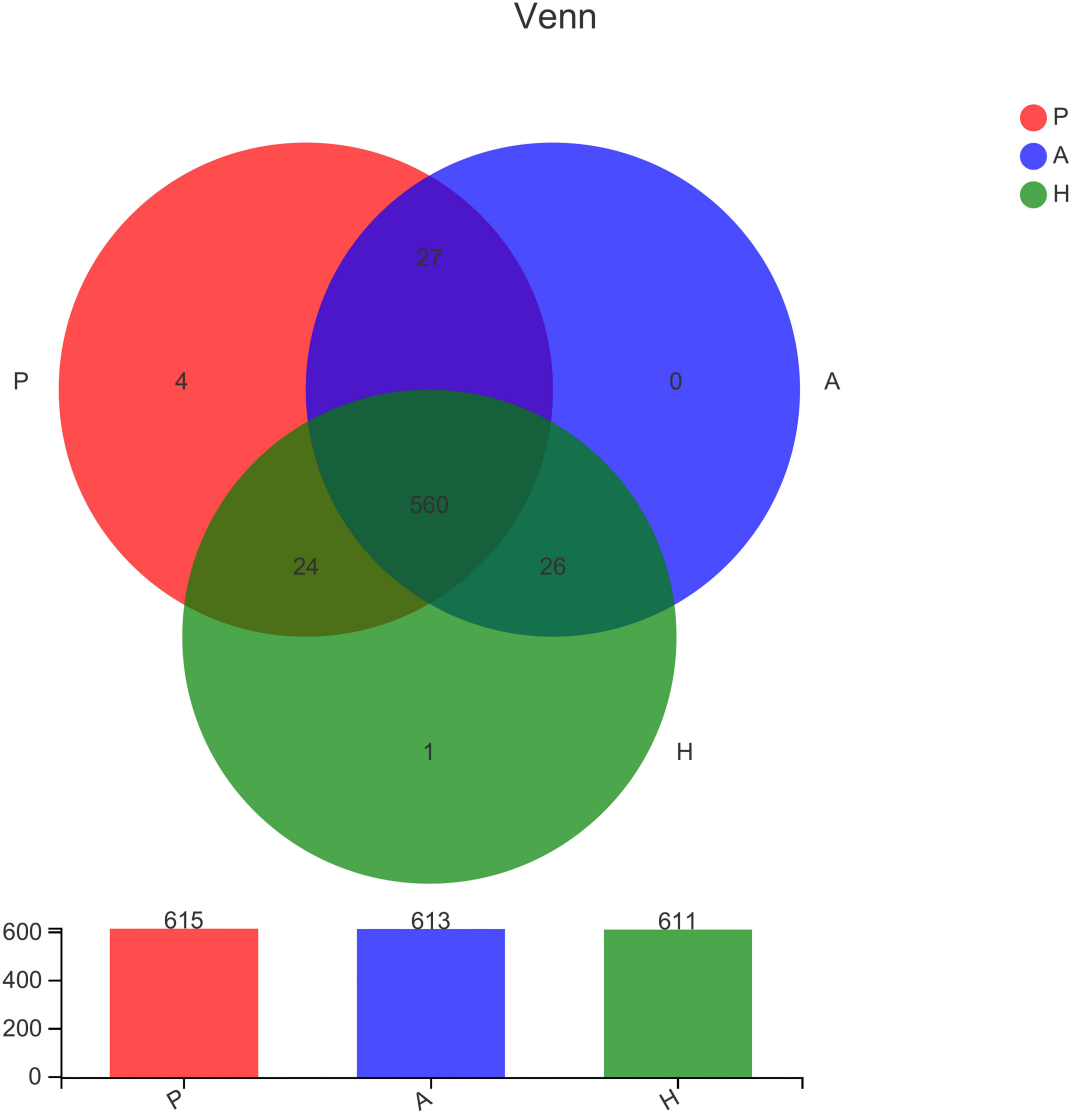
Comparison of the type and number of operational taxonomic units. In the Venn diagram, rare microbial operational taxonomic units (OTUs) were removed without subsampling. There was a lot of overlap in the OTUs of each group. Group P, patients with Peutz–Jeghers syndrome (PJS); group A, asymptomatic relatives; group H, healthy controls.

16S sequencing was used to analyze the bacterial fraction of the samples and assess the extent of microbiota dysbiosis related to PJS. The estimate of coverage reached >99.9% in all samples. After rare OTUs were removed, 11 phyla, 15 classes, 31 orders, 61 families, 159 genera, 313 species, and 498 OTUs were obtained for further analysis. The alpha-diversity of the bacteria was estimated and is shown in Figure 2. Flora of patient with PJS had a slightly reduced Chao- and ACE-estimated microbial richness, but this did not differ significantly from the AR and HC flora (Supplementary Table 1). However, using the Shannon indices, the AR and HC groups had higher microfloral diversity than the PJS group (*p* < 0.05). Similarly, the Simpson indices showed that the microflora in the AR and HC groups was more diverse than that in the PJS group (Wilcoxon rank-sum test, *p* < 0.01). There were no significant differences in microbial diversity between the AR and HC groups. Our result suggests that the bacterial alpha-diversity was diminished in patients with PJS, but was very similar between the ARs and HCs. A Venn diagram of the OTUs in 64 samples indicated the number of common and unique species (Figure 3). The PJS, AR, and HC samples had 560 OTUs in common, suggesting that the microbial composition was similar across all groups.

### Fecal Samples of Patients With PJS Reveal an Altered Bacterial Microbiome Structure

**Figure 4:**
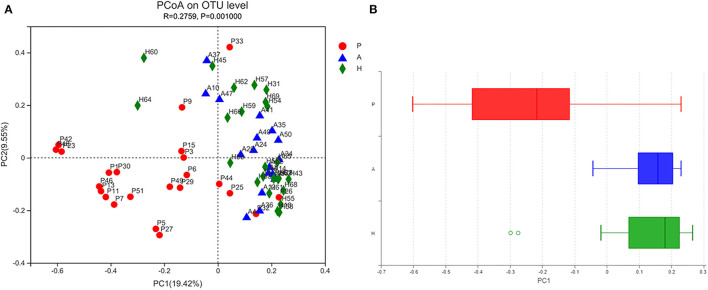
Bacterial microbiota biodiversity. **(A)** Principal coordinates analysis (PCoA) of the microbiota among the Peutz–Jeghers syndrome (PJS), AR, and HC groups (group P, patients with PJS; group A, asymptomatic relatives; group H, healthy controls). **(B)** Box plots of beta diversity in patients with PJS (red), asymptomatic relatives (blue), and healthy controls (green).

**Figure 5:**
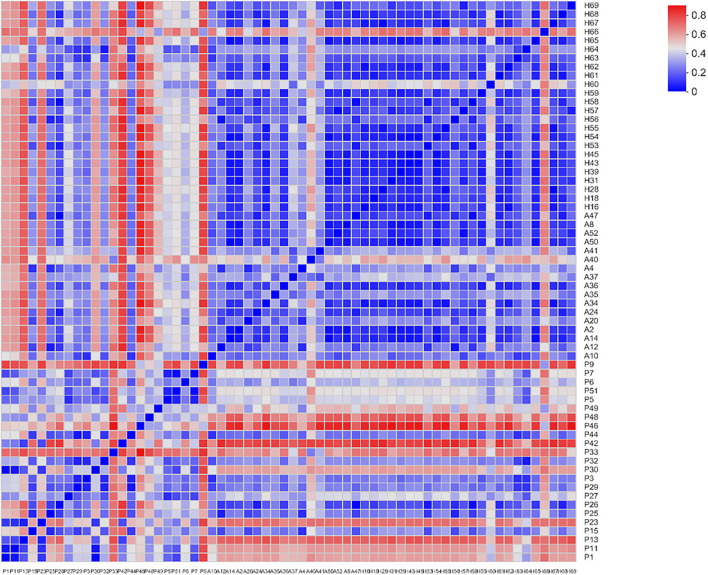
Sample distance heatmap at the phylum level.

Beta-diversity was evaluated using PCoA, which showed significant differences in the clustering of specimens by group phenotype. However, there was no clear clustering between the AR and HC groups (ANOSIM, *R* = 0.2759, *p* = 0.001). While the samples were aggregated in the PJS group, the distance between these samples was relatively more diffuse in the other groups (Figure 4). The PJS group showed significantly different bacterial beta-diversity than the AR and HC groups. In a sample distance heatmap at the phylum level, the distribution was uniform in the AR and HC groups (Figure 5). Thus, the species were highly similar among the samples and exhibited favorable clustering.

### Bacterial Abundance in Samples of Patients With PJS

**Figure 6:**
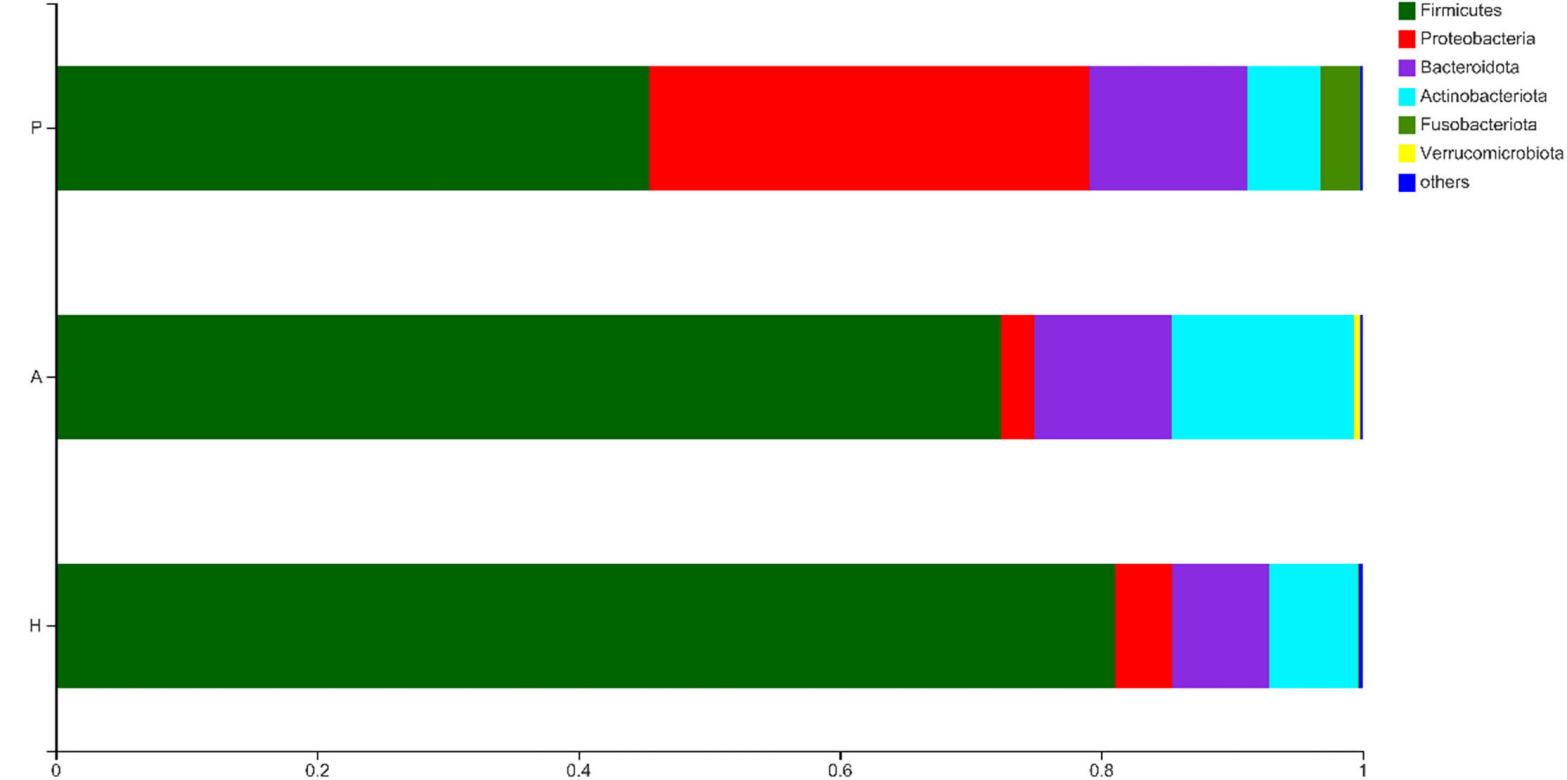
Community barplot analysis of bacteria at the phylum level.

The OTUs were compared with the database, and a bar plot and pie chart were created at the phylum, class, order, family, genus, and species levels. Six phyla, namely, Firmicutes, Proteobacteria, Bacteroidota, Actinobacteriota, Fusobacteriota, and Verrucomicrobiota were the most predominant (other phyla represented <0.005%). The results of the principal component distribution are shown in Figure 6. The abundance of Firmicutes (45.45%) and Actinobacteriota (5.65%) was significantly lower in the PJS samples than in the AR and HC samples, while the abundance of Proteobacteria (33.67%) and Fusobacteriota (3.03%) was significantly higher in the PJS samples.

At the class level, the PJS group primarily included Clostridia (36.88%), Gammaproteobacteria (33.67%), and Bacteroidia (12.01%). The abundance of Clostridia was significantly lower, while the abundance of Gammaproteobacteria was higher in the PJS group than in the other groups (*p* < 0.05). At the order level, the primary bacteria in the PJS group were Lachnospirales (17.40%), Oscillospirales (17.25%), Enterobacterales (33.56%), Bacteroidales (12.01%), Lactobacillales (6.36%), Bifidobacteriales (4.14%), and Fusobacteriales (3.03%). The abundance of Enterobacterales was significantly higher, and the levels of Lachnospirales and Oscillospirales were lower in the PJS group than in the other groups. At the family level, the predominant bacteria in the PJS samples included Lachnospiraceae (17.40%), Ruminococcaceae (13.94%), and Enterobacteriaceae (33.53%). The abundance of Enterobacteriaceae was significantly higher in the PJS group than in the other groups. The abundance of Lachnospiraceae and Ruminococcaceae was markedly lower in the PJS group than in the other groups (*p* < 0.05). At the genus level, the abundance of *Escherichia-Shigella* (27.54%) and *Bacteroides* (7.58%) was significantly higher in the PJS group than in the other groups (*p* < 0.05), but the abundance of *Faecalibacterium* (8.43%), *Blautia* (4.24%), *Bifidobacterium* (4.14%), *Subdoligranulum* (3.42%), and *Megamonas* (0.57%) was slightly lower in the PJS group than in the other groups. At the species level, the abundance of *Escherichia coli* of *Escherichia-Shigella* (27.34%) was markedly higher in the PJS group (*p* < 0.05), while the abundance of *Faecalibacterium prausnitzii* (7.92%), uncultured bacteria of *Subdoligranulum* (3.34%), unclassified *Blautia* (2.63%), and *Eubacterium pseudocatenulatum* (2.58%) was lower than in the other groups. A community bar plot analysis of the different bacterial levels is shown in Supplementary Figure 1.

### Metagenome Biomarkers of Bacterial Flora in Patients With PJS

**Figure 7:**
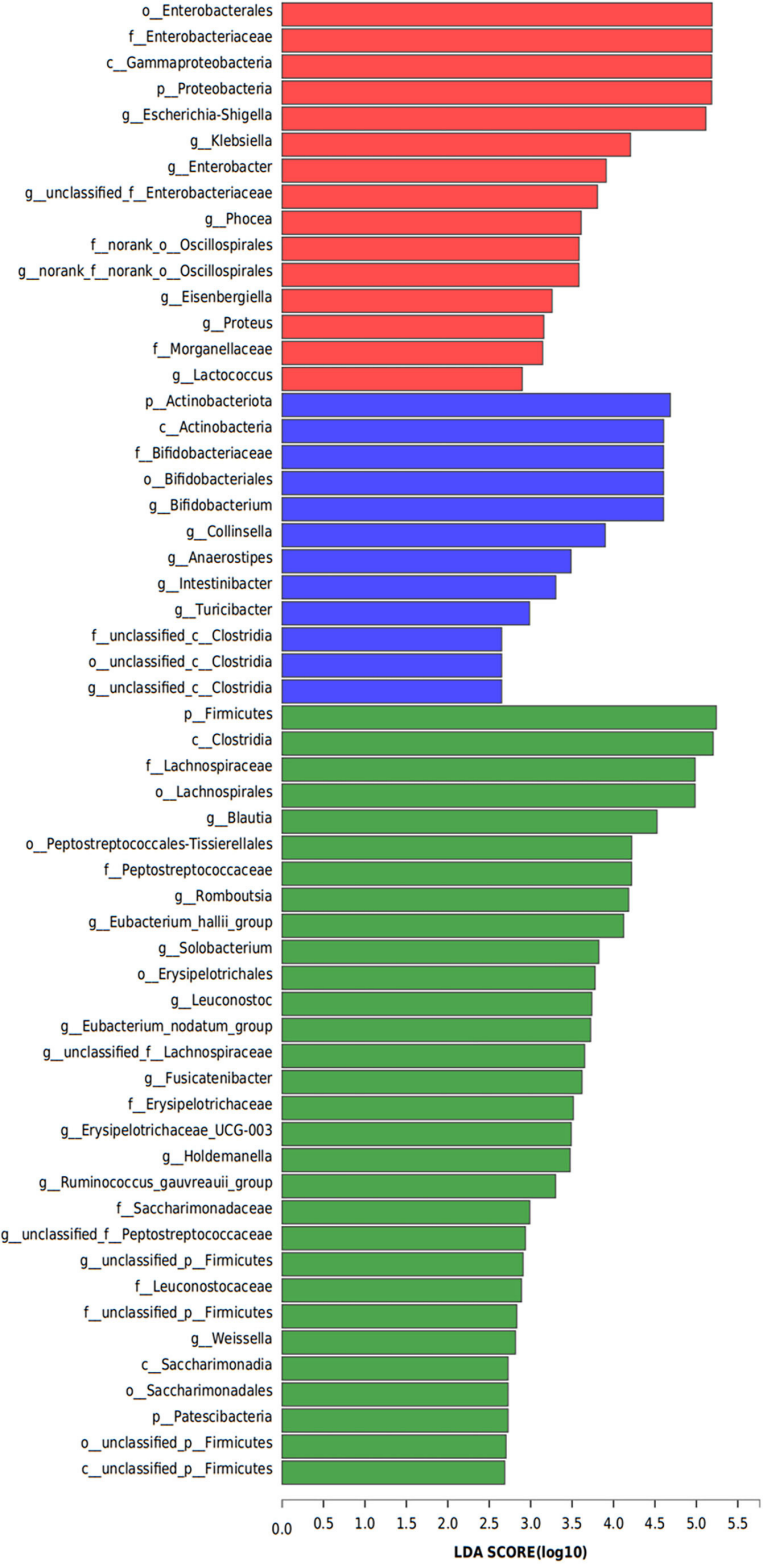
Metagenome biomarkers of bacterial flora. Analysis of metagenome biomarkers with linear discriminant analysis of effect sizes (LEfSe). Important taxonomic differences in the fecal microbiomes were identified among the three groups (LDA > 2, non-parametric factorial Kruskal–Wallis rank-sum test, *p* < 0.05).

Linear discriminant analysis (LDA) effect size was used to identify the differences in metagenome biomarkers between the three groups. The biomarkers in PJS group were Enterobacterales (order), Enterobacteriaceae (family), Gammaproteobacteria (class), Proteobacteria (phylum), and *Escherichia-Shigella* (genus). The abundance of Actinobacteriota (phylum), Actinobacteria (class), Bifidobacteriaceae (family), Bifidobacteriales (order), and *Bifidobacterium* (genus) was higher in the AR group while the abundance of Firmicutes (phylum), Clostridia (class), Lachnospiraceae (family), Lachnospirales (order), and *Blautia* (genus) was significantly higher in the HC group than in the other groups (Figure 7).

### Diversity of the Fungal Microbiota in Patients With PJS

**Figure 8:**
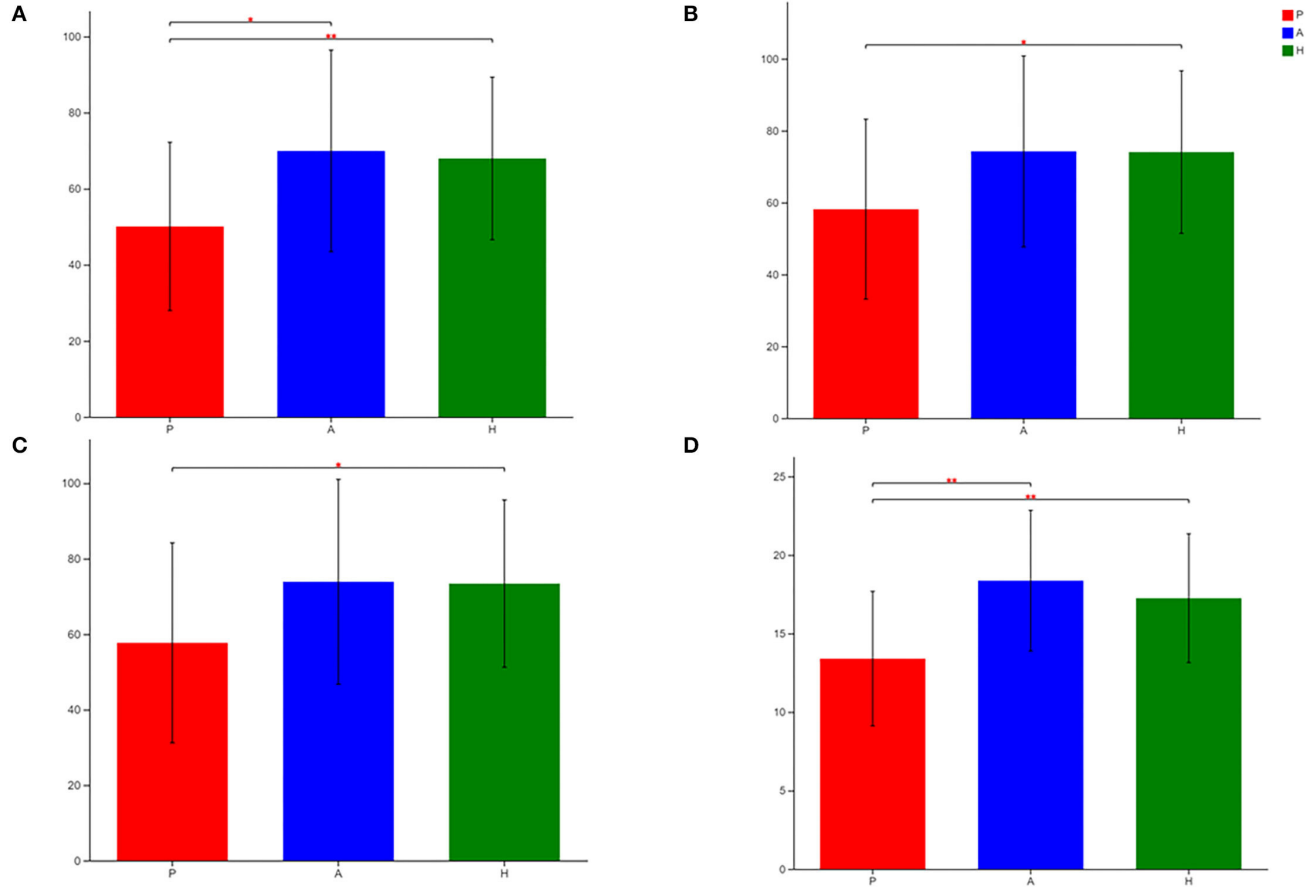
Differences in sobs **(A)**, ace **(B)**, Chao **(C)**, and pd **(D)** of fungal flora among patients with Peutz–Jeghers syndrome (PJS) (group P), asymptomatic relatives (group A), and healthy controls (group H) at the OTU level (Student's *t*-test, *p < 0.05; **p < 0.01).

**Figure 9:**
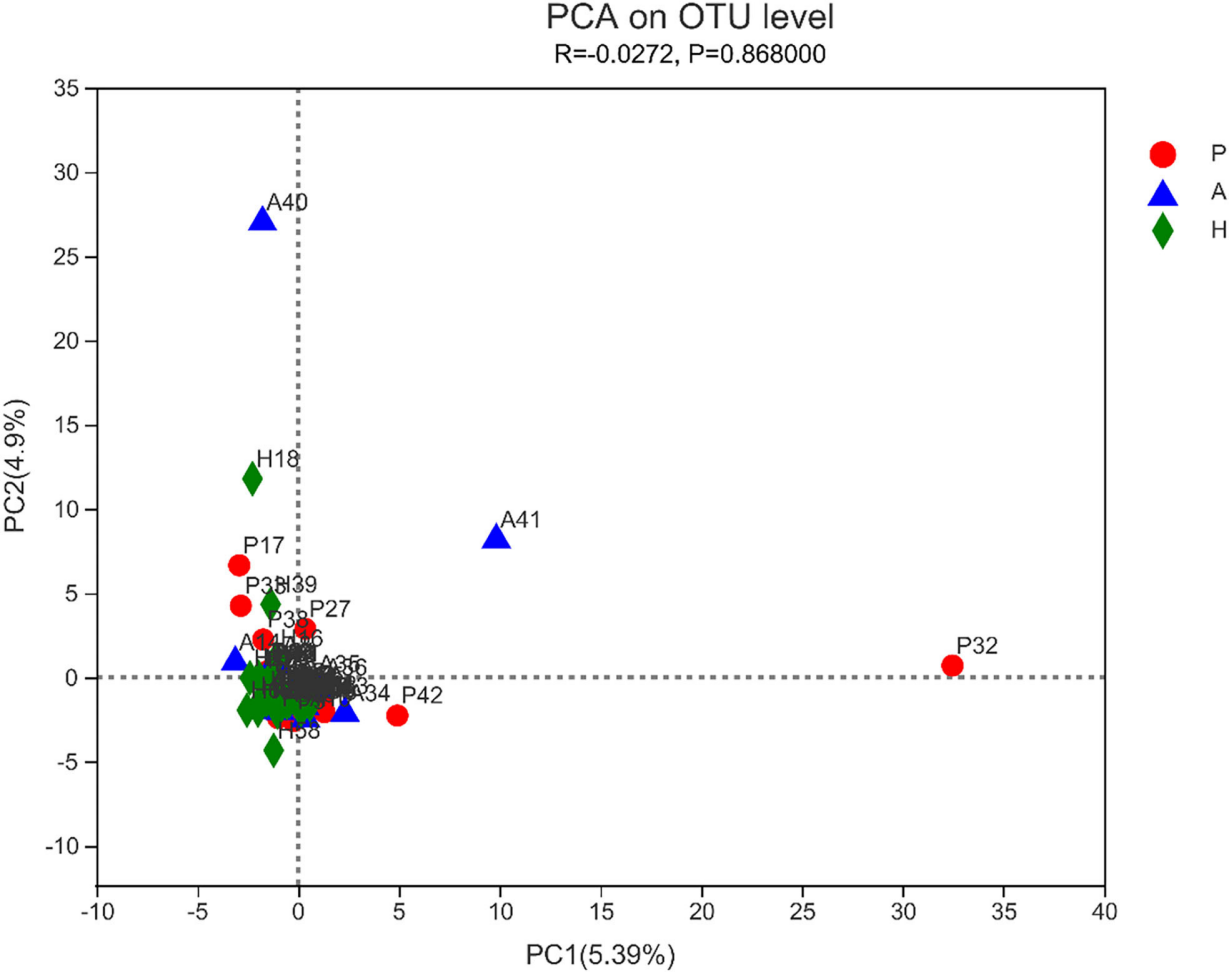
Beta-diversity of fungi was determined using principal component analysis (PCA). There was no obvious clustering of samples by groups.

**Figure 10:**
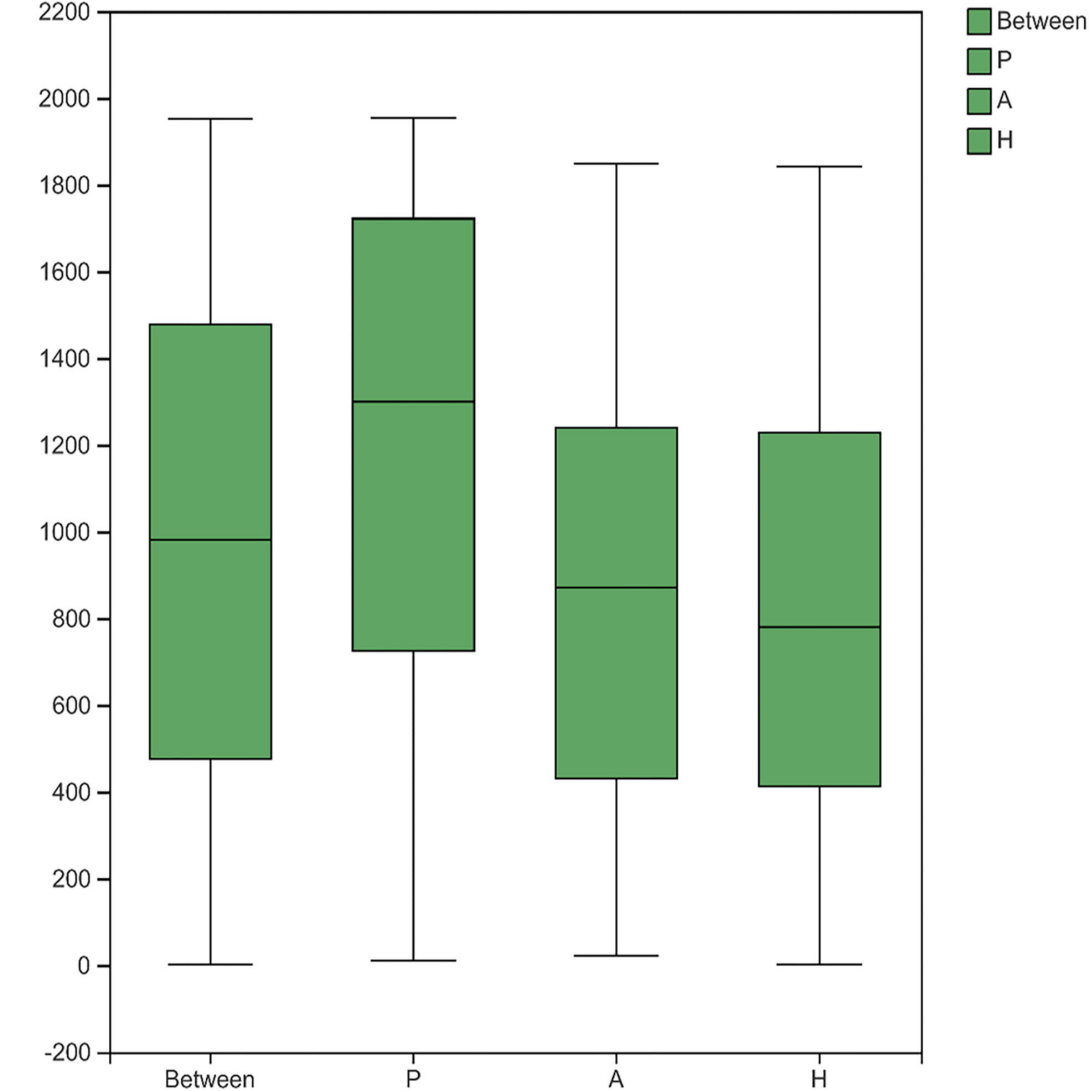
Analysis of similarities (ANOSIM/Adonis) between the fungi. The ordinate represented the rank of distance calculated on the operational taxonomic unit (OTU) level of each group.

The sobs, ace, pd, and Chao indices at the OTU level showed that patients with PJS had less alpha-diversity than controls (Figure 8). However, the Shannon, Simpson, and coverage indices of fungal alpha diversity in patients with PJS were similar to those of HCs (*p* > 0.05). These indices also showed similar biodiversity between the AR and HC groups (Supplementary Table 2). There were no significant differences in the beta-diversity of the mycobiome in the PJS, AR, and HC groups (Figure 9). The analysis of similarities showed no significant distance calculated at the OTU level for each sample (ANOSIM/Adonis, Figure 10; *p* = 0.262).

### Composition and Structure of the Fungal Microbiota in Patients With PJS

**Figure 11:**
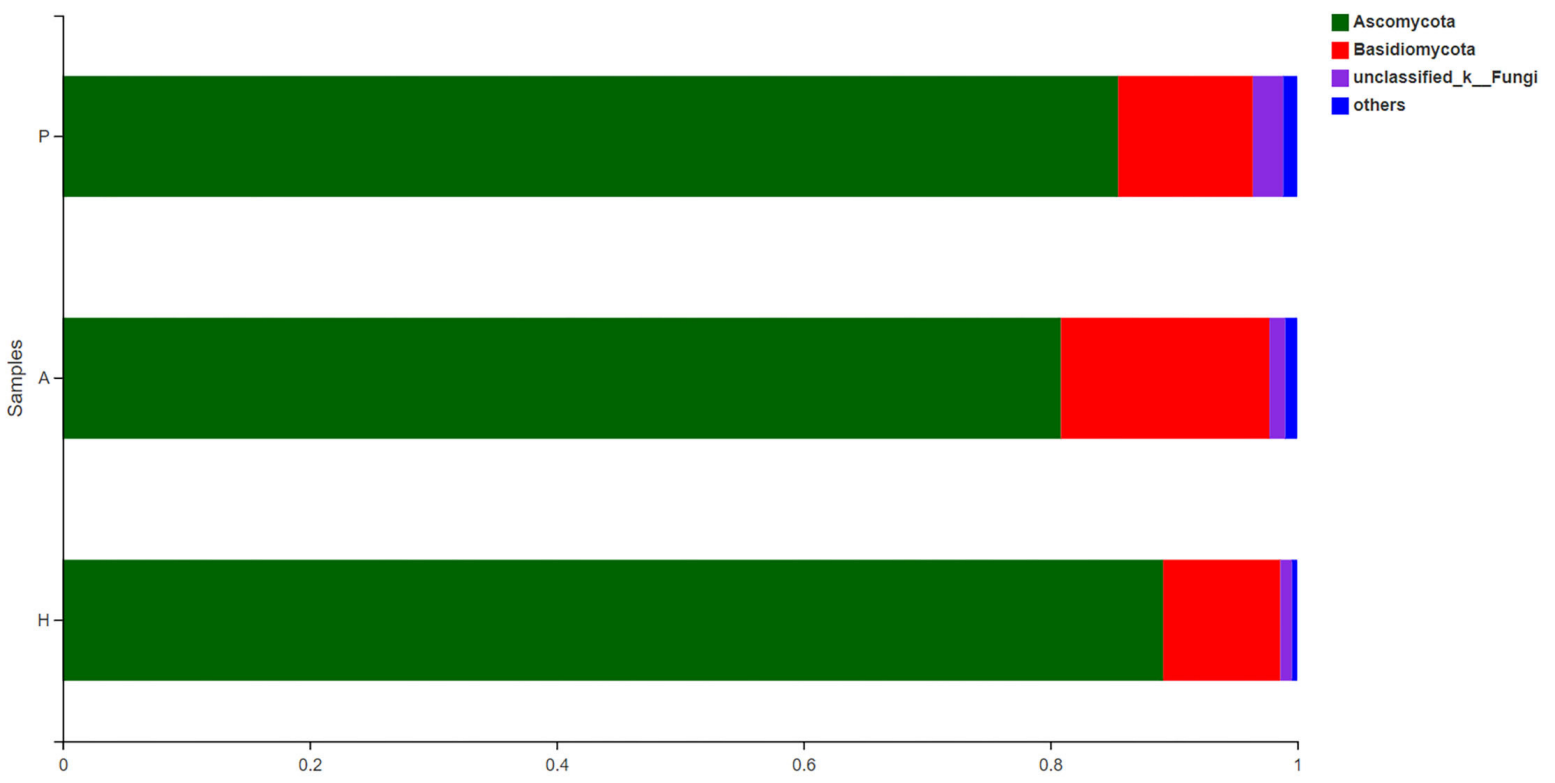
Composition of fungi at the phylum level.

The fecal fungal microbiota was dominated by two phyla, Ascomycota and Basidiomycota, in all groups (Figure 11). At the phylum level, there was a higher proportion of unclassified fungal phyla in the PJS group than in the HC group. In all samples, Eurotiomycetes and Saccharomycetes were the dominant classes of the Ascomycota phylum, and Tremellomycetes was the dominant class of the Basidiomycota phylum. Cross-sectional analysis revealed no significant differences in the Basidiomycota/Ascomycota ratio, a measure of fungal dysbiosis, while the HC group had the lowest ratio.

**Figure 12:**
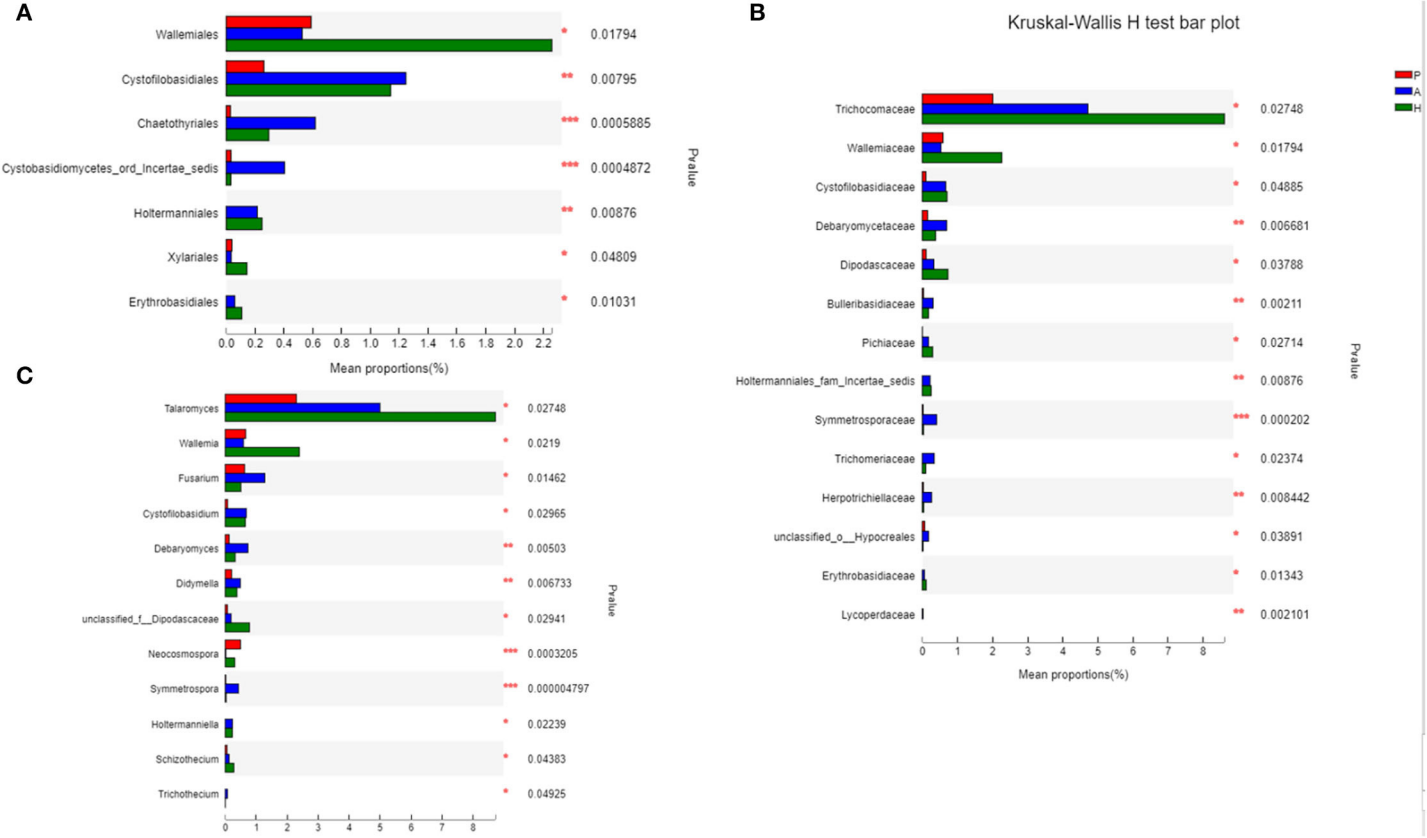
Biomarkers of fungi in patients with Peutz–Jeghers syndrome (PJS) at different levels. **(A)** Biomarkers in PJS samples at the order level (Kruskal–Wallis *H*-test). **(B)** Biomarkers in PJS samples at the family level (Kruskal–Wallis *H*-test). **(C)** Biomarkers in patients with PJS at the genus level (Kruskal–Wallis *H*-test). *p < 0.05; **p < 0.01; ***p < 0.001.

**Figure 13:**
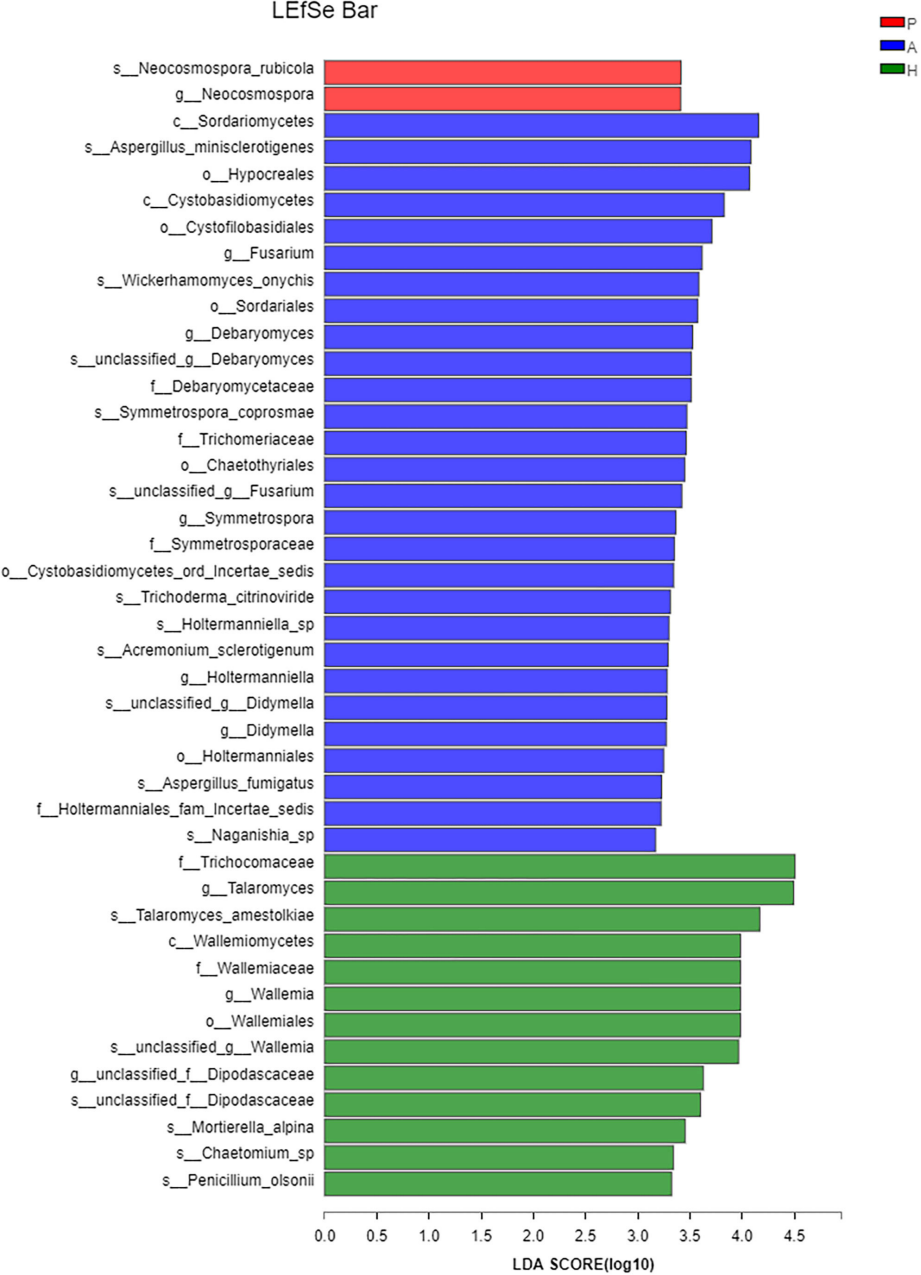
Linear discriminant analysis of effect sizes (LEfSe) barplot of fungal flora. Linear discrimination analysis (LDA) was used to estimate the effect of abundance of each component on the differential effect.

**Figure 14:**
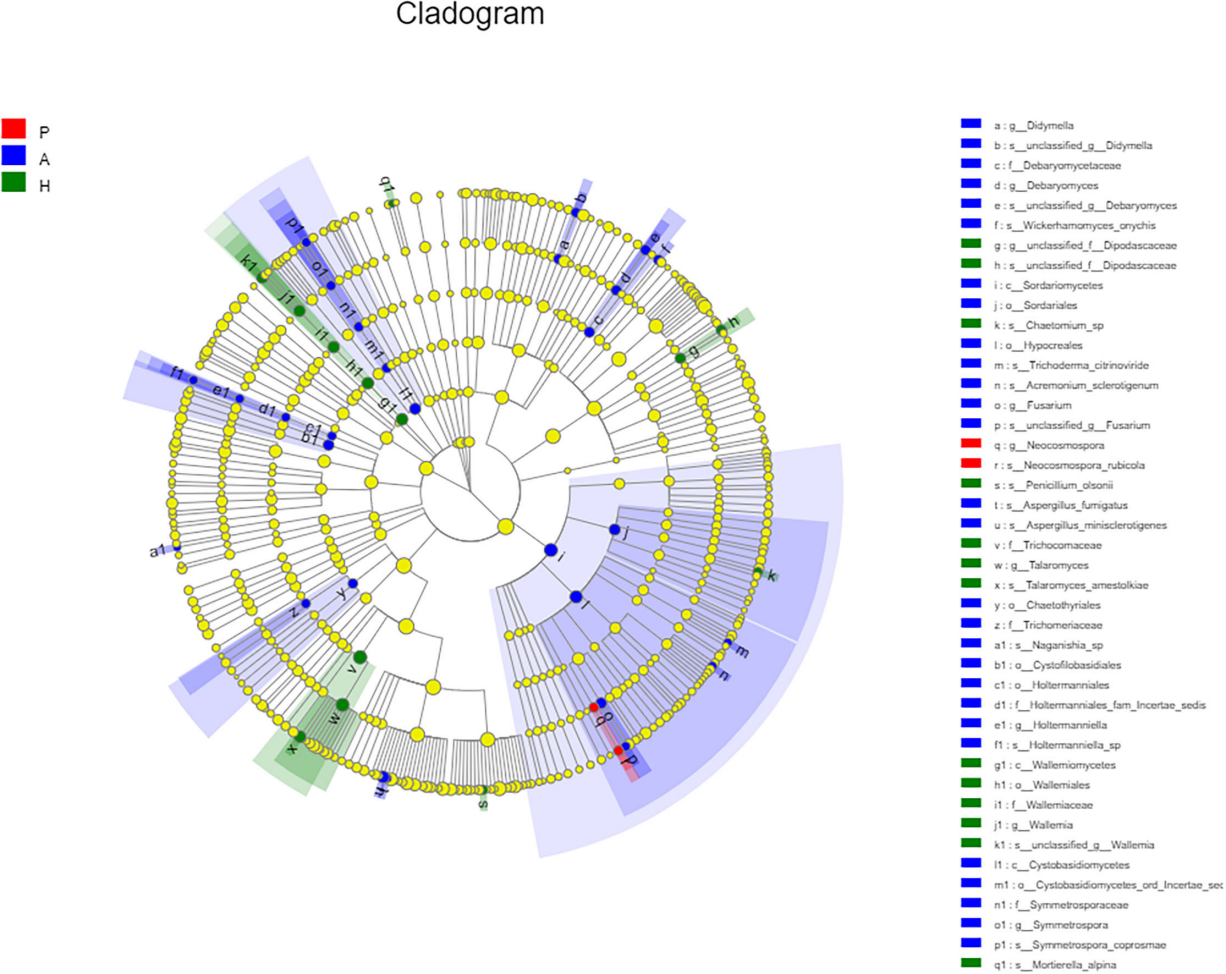
Cladogram of fungal flora. Nodes with different colors indicate microbial groups that were enriched and show significant differences among the groups. Yellow nodes indicate microbial groups without significant differences among the groups. The size of the circle in the cladogram was proportional to fungal abundance. Moving from the inside to the outside, the circles represent the phylum, class, order, family, and genus of fungi, respectively.

At the class level, lower levels of Eurotiomycetes and higher levels of Saccharomycetes were observed in the PJS group. At the order level, the abundance of Eurotiales was lower in the PJS group. However, these differences did not reach statistical significance. At the genus level, *Aspergillus, Penicillium*, and *Candida* were present in all groups, and the abundance of *Candida* was higher in the PJS group than in the other groups. At the species level, *Candida albicans* and *Cutaneotrichosporon curvatus* were higher in the PJS group (Supplementary Figure 2). Importantly, unclassified fungi accounted for most sequences at the species level, suggesting that the current fungal gene databases are incomplete. However, the Kruskal–Wallis *H*-test and one-way ANOVA did not show significant differences at the phylum level. Some fungal species also had significantly different levels of abundance among the groups (class, order, family, genus, and species levels), including the Cystofilobasidiales order, the Trichocomaceae family, and the *Talaromyces* genus (Figures 12A–C; *p* = 0.02748). The LEfSe plots and cladograms showed the species of fungi with the largest differences in relative abundances (Figures 13, 14). The *Neocosmospora rubicola* and *Neocosmospora* genera were significantly higher in the PJS group than in the other groups (LDA = 2).

### The Predicted Microbial Functions That Were Altered in Patients With PJS

The functional composition profiles from 16S rRNA sequencing data were predicted using PICRUSt to characterize functional alterations in the gut microbiome of patients with PJS. Several KEGG (module-level) categories were lower in the PJS samples (Supplementary Figure 3). The Embden–Meyerhof pathway (M00001), gluconeogenesis (M00003), histidine biosynthesis and PRPP pathway (M00026), lysine biosynthesis and succinyl-DAP pathway (M00016), energy metabolism (M00157), and inosine monophosphate biosynthesis (M00048) were particularly enriched in control participants. Meanwhile, the reductive citrate cycle (Arnon-Buchanan cycle, M00173) and citrate cycle (TCA cycle, Krebs cycle, M00009) were enriched in the PJS group. Fungal OTUs were assigned to specific functional groups using the FunGuild database. The guilds identified in this study are listed in Supplementary Figure 4. Undefined saprotrophs accounted for ~70% of all fungal OTUs detected. The relative abundance of undefined saprotrophs was higher in the control group than in the other groups, and animal pathogens were higher in the PJS group than in the other groups. Overall, these findings indicated that shifts in microbial community composition might interfere with physiological processes in the host.

## Discussion

While studies have characterized the enteric microflora contributing to the pathogenesis of several digestive diseases, the relationship between enteric microflora and PJS remains unknown. This study was the first to compare the bacterial and fungal fecal flora of patients with PJS with the flora of their relatives and HCs. Patients with PJS and HCs had different abundances, structures, and compositions, suggesting a role for microbes in disease pathogenesis.

An analysis of beta-diversity showed that the fecal microbiota of the PJS group had clustering that was distinct from the AR or HC groups, while the fecal microflora of the HC and AR groups clustered together. Significant distinctions were found between patients and their relatives with similar diets and genetics, suggesting that alterations of the microbiota may contribute to PJS pathogenesis or progression. This was confirmed using alpha-diversity analysis, which showed a decrease in biodiversity in the PJS group and less difference in diversity between the AR and HC groups. Major differences in bacterial composition were also observed, including a clear reduction in Firmicutes and an increase in Proteobacteria and Fusobacteria in patients with PJS. The alterations and decreased biodiversity were similar to microbiota studies of IBD (Xu et al., 2018), CRA (Feng et al., 2015), CRC, colitis-associated cancer (CAC), sporadic cancer (SC) (Mima et al., 2016; Richard et al., 2018; Wang et al., 2020), PTEN hamartoma tumor syndrome (PHTS) (Jia et al., 2021), and familial adenomatous polyposis (Dejea et al., 2018). The data were obtained from fecal and mucosal samples, suggesting that potential similarities were present in the fecal and mucosal flora of patients with PJS. Further study of mucosal samples is necessary. Interestingly, patients with non-gastrointestinal cancers, such as pancreatic adenocarcinoma (Ren et al., 2017; Thomas and Jobin, 2020) and breast cancer (Goedert et al., 2015; Chen et al., 2019; Byrd et al., 2021), had similar changes in their microbiome.

At the family level, Lachnospiraceae and Ruminococcaceae were found at a lower level in the PJS group than in the other groups. Lachnospiraceae mitigates inflammation and carcinogenesis of the bowel and suppresses *Clostridium difficile* colonization in patients who are on antibiotic therapy (Reeves et al., 2012; Wang et al., 2020). Some members of the Ruminococcaceae family produce secondary bile acid (SBA) (Sinha et al., 2020). The lack of SBA is associated with intestinal inflammation, as shown by the diminished levels in patients with IBD (Theriot et al., 2016). Thus, the lower levels of Lachnospiraceae and Ruminococcaceae might correlate with the increased gut mucosal inflammation observed in patients with PJS. Enterobacteriaceae, Streptococcaceae, and Fusobacteriaceae were significantly higher in this patient population. Enterobacteriaceae, which includes commensal organisms, primary pathogens, and opportunistic pathogens, are often abundant in the inflamed gut (Sassone-Corsi et al., 2016). Streptococcaceae, from phylum Firmicutes, have potential oncogenic effects (Abdulamir et al., 2011) and are directly associated with both metabolic syndrome and colon cancer (Aran et al., 2011; Qiao et al., 2014; Zeng et al., 2016).

At the genus level, patients with PJS had significantly higher levels of *Escherichia-Shigella* than the AR and HC groups. *Escherichia-Shigella* is associated with mucosal inflammation and IBD pathogenesis (Xu et al., 2018). Lipopolysaccharide from *Escherichia-Shigella* genus can activate Toll-like receptor 4 (Beutler, 2002) and the nuclear factor kappa B pathway and induce IL-1, IL-6, and tumor necrosis factor-α production (Zhou et al., 2020), causing inflammatory and oxidative damage (Lamping et al., 1998; Vonlaufen et al., 2014). *Streptococcus* was also found in higher levels in patients with PJS, which was similarly elevated in CAC tumor sites (Richard et al., 2018). *Streptococcus* and *Escherichia-Shigella* are human pathogens that can be risk factors for ulcerative colitis progression (Dai et al., 2020; Jialing et al., 2020). The severity of inflamed mucosa correlates positively with the abundance of these bacteria in IBD. There were a significant abundance of several other genera, including *Bacteroides, Klebsiella*, and *Fusobacterium*, in patients with PJS and a decrease in *Blautia, Subdoligranulum*, and *Megamonas*. *Bacteroides* are also increased in patients with IBS (Pittayanon et al., 2019) and may contribute to the inflammatory environment. Indeed, some enterotoxigenic *Bacteroides* strains can produce toxins (Merino et al., 2011; Le Chatelier et al., 2013) and cause abdominal pain and diarrhea (Macfarlane et al., 2005). *Klebsiella* is often observed in patients with gastrointestinal diseases (Kaur et al., 2018). These bacteria may play an important role in CRC progression by stimulating epithelial cell proliferation. There is also a positive association between *Fusobacterium*, particularly *Fusobacterium nucleatum*, and CRC. *Fusobacterium* can cause opportunistic infections (Brennan and Garrett, 2019), and some studies have suggested that it may also have a carcinogenic effect by activating inflammatory and oncogenic pathways and regulating the tumor immune environment (Li et al., 2016; Nosho et al., 2016; Hussan et al., 2017). *Fusobacterium* abundance is higher in PHTS cases than in controls (Jia et al., 2021). *Blautia* abundance is also lower in patients with CRC than in controls (Richard et al., 2018). *Subdoligranulum*, which support intestinal health by producing butyrate, providing host cells with energy, and maintaining the integrity of the gut barrier, are found in lower abundances in patients with IBD (Thomas et al., 2014; Deaver et al., 2018; Zhou et al., 2020). *Blautia* and *Subdoligranulum* are reduced in inflammation-associated microbiota, indicating that the PJS microenvironment is unfavorable for bacterial strains that play an anti-inflammatory role, thus, indirectly promoting polyp or tumor development. Few studies have assessed the role of *Megamonas*, a vital acetate- and propionate-producing genus, in the gut (Zhang et al., 2013; Chen et al., 2014; Polansky et al., 2015; Zhao et al., 2019). Thus, further research is necessary to determine its potential impact on gastrointestinal health. At the species level, *E. coli*, from the *Escherichia-Shigella* genus, and *Klebsiella pneumoniae* were higher in PJS samples than in AR and HC samples, suggesting a role for *E. coli* in disease pathogenesis. The colibactin toxin released from *K. pneumoniae* is shown to cause DNA strand breaks, which induce genomic instability and chronic inflammation (Holden et al., 2016); however, there were still many uncultured or unclassified *Klebsiella* species with unknown functions. Current technologies and databases are not yet comprehensive enough to study microbial functions in full detail.

The fecal bacterial microbiota in patients with PJS had low biodiversity and abnormal proliferation of some taxa related to opportunistic pathogens, inflammation, and carcinogenesis. Fungal flora tends to be regarded as a relatively minor part of the gut microbiome, accounting for roughly 0.1% of the microflora. The function of the mycobiome in the human gut remains largely unknown because fungi classifications are absent from current genomic databases (Sartor and Wu, 2017). In this study, alpha-diversity was lower in the PJS group, which was similar to the altered bacterial diversity observed in PJS samples. However, statistically significant indicators of biodiversity were not enough. There were fewer differences in the composition of fungal flora in the studied groups than in the composition of bacterial flora, and individual differences were greater. A few notable trends included the increased proportions of *Candida* (genus level) and *C. albicans* (species level) and a higher Basidiomycota/Ascomycota ratio in PJS samples. Interestingly, Sokol et al. have also observed an increased proportion of *C. albicans* and a higher Basidiomycota/Ascomycota ratio in the fecal fungal microbiota of patients with IBD than in HCs (Sokol et al., 2017). A higher *Candida* abundance has also been described in patients with PHTS (Jia et al., 2021). Thus, imbalanced fungal diversity and abundance might also contribute to the inflammatory process, or polyp formation in patients with PJS. An abundance of certain bacterial or fungal taxa could be a key indicator of immune dysfunction associated with tumor or polyp formation during PJS.

While the changes in fungal abundance were small, fungal diversity also showed a decreasing trend. Thus, there might be a correlation between bacteria and fungi during disease development. Compared with bacteria, alterations in fungal abundance were not significant, potentially because (1) the sample size was insufficient, (2) the same amount of feces contained far fewer fungi than bacteria, and (3) the fungal database and current detection technologies are still incomplete. Additional studies using larger sample sizes and more advanced detection techniques are required to define the role of fungi and the relationship between bacteria and fungi in PJS development and progression. Fewer studies on fungi have been conducted, but those that have assessed bacteria and fungi together have shown fewer changes in fungal abundance (Richard et al., 2018; Jia et al., 2021).

Geography, diet, and race influence the composition of intestinal bacterial and fungal flora (Escobar et al., 2014; Graf et al., 2015). The human intestinal fungal composition is individual-specific. This study recruited healthy family members as controls to alleviate the influence of hereditary, dietary, geographical, and racial factors on the results (Song et al., 2013). Additionally, all participants in this study were of Chinese Han ethnicity, mitigating the influence of ethnicity. The impact of sex and age on floral composition among the groups was also assessed but was not statistically significant (*p* > 0.05). The microbiota characteristics in first-degree ARs were grouped in-between those from patients with PJS and HCs. However, these differences in the composition and structure of gut flora between the AR and HC groups did not reach statistical significance, suggesting that diet, lifestyle, consanguinity, and other factors did not have an obvious impact on the fecal flora of healthy individuals.

For the first time, biomarkers of PJS were screened in this study. With the development of intestinal microecological detection technology, the significantly reduced biodiversity and specific bacterial composition associated with PJS could be used to aid diagnosis and cancer monitoring. Many patients worried that children had their condition, and some relatives of patients with PJS were asymptomatic, merely with skin or mucosa hyperpigmentation. Improved microecological detection technologies may allow avoiding enteroscopy. The assessment of stool samples is safe and more convenient and less expensive than colonoscopies.

Understanding the regulatory functions of microbes in the gut could help characterize the pathogenesis of PJS and establish individualized strategies to retouch the enteric microflora of patients with PJS. Patients with PJS requiring regular enteroscopy and gastrointestinal mucosal hamartomatous polyp removal experience significant discomfort. In recent years, probiotics have been used to modulate the gut microbiota of individuals with a number of different diseases. The role of the selective microbiota in PJS development and progression requires further investigation, possibly by transferring PJS microbiota or key species into germ-free mice. Animal models could address whether the removal of particular bacteria or fungi successfully slows disease progression and provide new treatment approaches for patients with PJS. It is necessary to evaluate whether microecological agents (such as prebiotics, probiotics, and personalized fecal transplantation) are effective in slowing the progression of polyps. Further research on microbiota-related metabolic pathways and immune molecules is required to identify additional therapeutic targets. Characterizing the relationship between intestinal flora and inflammation could also help design new therapies aimed at controlling PJS inflammation and polyp growth.

This study had several limitations. First, this investigation should be followed by a larger-scale study with larger samples. Second, stool samples provide incomplete intestinal flora, and the intestinal microbiota includes both intestinal flora from the lumen and mucosa-related microflora (Chen et al., 2012). Data specific to the mucosal microbiota flora of patients with PJS are also essential for future research. Luminal microbes will need to be compared with mucosa-associated microbes, including whether specific taxa associated with the mucosa can influence the development of PJS. Third, it was difficult to identify temporal associations and mechanisms of microbial dysbiosis in patients with PJS because this study was cross-sectional. Fourth, experiments based on animal models were absent in our research, which is necessary for the study of mechanisms that link microbiota dysbiosis to PJS development.

The role of the enteric microflora in PJS pathogenesis remains unknown. This study provided initial evidence that dysbiosis of fecal microbiota diversity and composition were significant in patients with PJS. This is the first study to compare the bacterial and fungal fecal flora of patients with PJS with the flora of their relatives and HCs, and characterize the microbiota profile of patients with PJS, which may provide a basis for further microecology research on PJS.

## Data Availability Statement

The data presented in the study are deposited in the NCBI Sequence Read Archive repository, accession numbers PRJNA802067 and PRJNA838192.

## Ethics Statement

The studies involving human participants were reviewed and approved by Medical Ethics Committee of Qilu Hospital of Shandong University. Written informed consent to participate in this study was provided by the participants' legal guardian/next of kin. Written informed consent was obtained from the individual(s), and minor(s)' legal guardian/next of kin, for the publication of any potentially identifiable images or data included in this article.

## Author Contributions

SW: literature search, figures, data analysis, data interpretation, and writing. GH, J-XW, and LT: study design and data collection. X-LZ, Y-QL, and Y-BY: funding acquisition, investigation, and project administration. All authors contributed to the article and approved the submitted version.

## Funding

This work was supported by the National Natural Science Foundation of China (NSFC 81670486 and 82070540), the National Key Research and Development Program of China (2017YFC0110003), and the Fundamental Research Fund of Shandong University (2017JC036).

## Author Disclaimer

The views expressed in this article are the authors' own and not an official position of the institution or funder.

## Conflict of Interest

The authors declare that the research was conducted in the absence of any commercial or financial relationships that could be construed as a potential conflict of interest.

## Publisher's Note

All claims expressed in this article are solely those of the authors and do not necessarily represent those of their affiliated organizations, or those of the publisher, the editors and the reviewers. Any product that may be evaluated in this article, or claim that may be made by its manufacturer, is not guaranteed or endorsed by the publisher.

## Supplementary Material

Supplementary Figure 1 Histograms of the different levels of bacteria. **(A)** Histograms on the class level. **(B)** Histograms on the order level. **(C)** Histograms on the family level. **(D)** Histograms on the genus level. **(E)** Histograms on the species level.

Supplementary Figure 2 Composition of fungi at different levels. **(A)** Composition of fungi at the class level. **(B)** Composition of fungi at the order level. **(C)** Composition of fungi at the family level. **(D)** Composition of fungi at the genus level. **(E)** Composition of fungi at the species level.

Supplementary Figure 3 Heatmap of module based on PICRUSt 2. Differences among patients with Peutz–Jeghers syndrome patients (Group P), asymptomatic relatives (Group A), and healthy controls (Group H).

Supplementary Figure 4 Variations in the composition of fungal functional groups inferred by FUNGuild. Differences in fungal functional groups among patients with Peutz–Jeghers syndrome (Group P), asymptomatic relatives (Group A), and healthy controls (Group H).

Supplementary Table 1 Indicators of α-diversity in bacteria. Peutz-jeghers syndrome (Group P), Asymptomatic relatives (Group A) and Healthy controls (Group H).

Supplementary Table 2 Indicators of α-diversity in fungal mycobiome. Peutz-jeghers syndrome (Group P), Asymptomatic relatives (Group A) and Healthy controls (Group H).
