# Supplementary material for: Altered Gut Microbiota in Patients With Peutz–Jeghers Syndrome
Source: Front Microbiol. 2022 Jul 13;13:881508. doi: 10.3389/fmicb.2022.881508 (PMC9326469; doi:10.3389/fmicb.2022.881508)
Supplement: Supplementary Figure 1 — Histograms of the different levels of bacteria. (A) Histograms on the class level. (B) Histograms on the order level. (C) Histograms on the family level. (D) Histograms on the genus level. (E) Histograms on the species level. [file Data_Sheet_1.zip › Table S2.DOCX]

**Supplementary Table 2. Indicators of α-diversity in fungal mycobiome**

| Estimators | A-Mean | P-Mean | H-Mean | Pvalue(A-P) | Pvalue(A-H) | Pvalue(H-P) |
| --- | --- | --- | --- | --- | --- | --- |
| sobs | 69.938 | 50.087 | 67.958 | 0.01537 | 0.7958 | 0.007181 |
| shannon | 2.6578 | 2.1364 | 2.5567 | 0.08231 | 0.6968 | 0.09048 |
| simpson | 0.18614 | 0.27995 | 0.20177 | 0.2197 | 0.8061 | 0.2382 |
| ace | 74.234 | 58.139 | 74.052 | 0.06176 | 0.9815 | 0.02684 |
| chao | 73.883 | 57.701 | 73.399 | 0.07115 | 0.951 | 0.03247 |
| coverage | 0.99933 | 0.99918 | 0.99915 | 0.3786 | 0.2522 | 0.8663 |
| pd | 18.364 | 13.398 | 17.248 | 0.001253 | 0.4215 | 0.002924 |

**NOTE**. Peutz-jeghers syndrome (Group P), Asymptomatic relatives (Group A) and Healthy controls (Group H)
