# Supplementary figures and images for: Altered Gut Microbiota in Patients With Peutz–Jeghers Syndrome
Source: Front Microbiol. 2022 Jul 13;13:881508. doi: 10.3389/fmicb.2022.881508 (PMC9326469; doi:10.3389/fmicb.2022.881508)

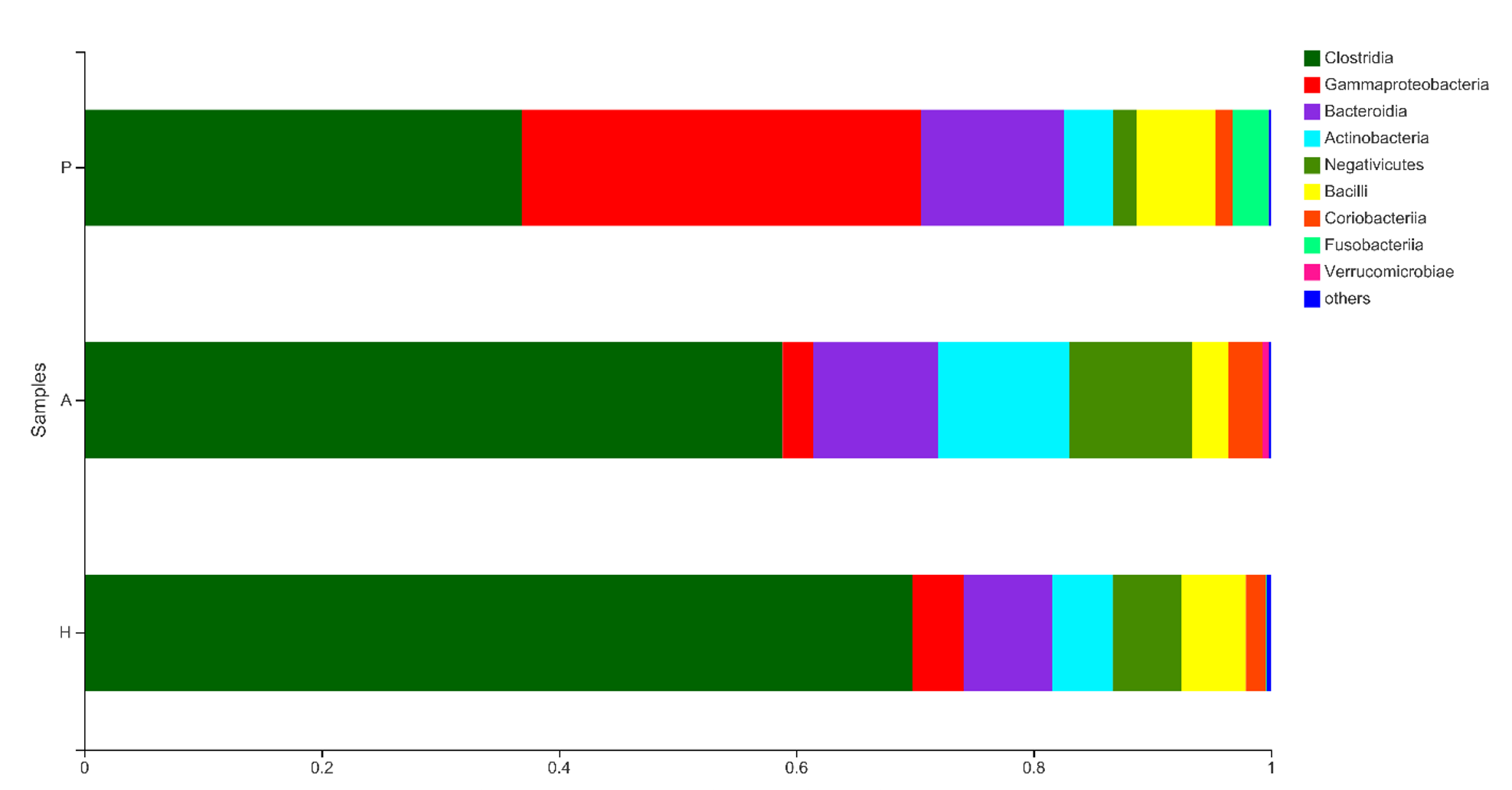

Supplement: Supplementary Figure 1 — Histograms of the different levels of bacteria. (A) Histograms on the class level. (B) Histograms on the order level. (C) Histograms on the family level. (D) Histograms on the genus level. (E) Histograms on the species level. [file Data_Sheet_1.zip › Figure S1A.TIF]

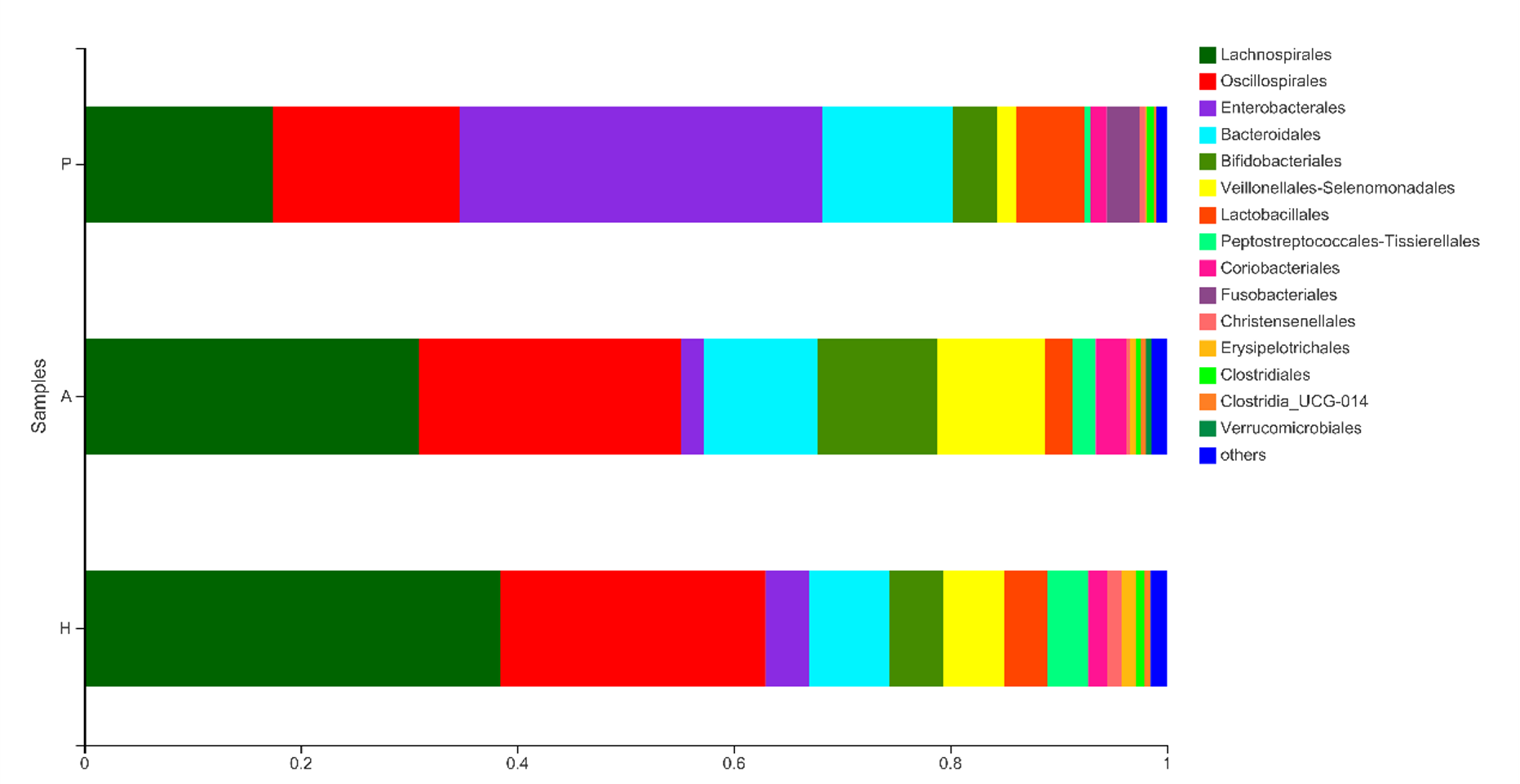

Supplement: Supplementary Figure 1 — Histograms of the different levels of bacteria. (A) Histograms on the class level. (B) Histograms on the order level. (C) Histograms on the family level. (D) Histograms on the genus level. (E) Histograms on the species level. [file Data_Sheet_1.zip › Figure S1B.TIF]

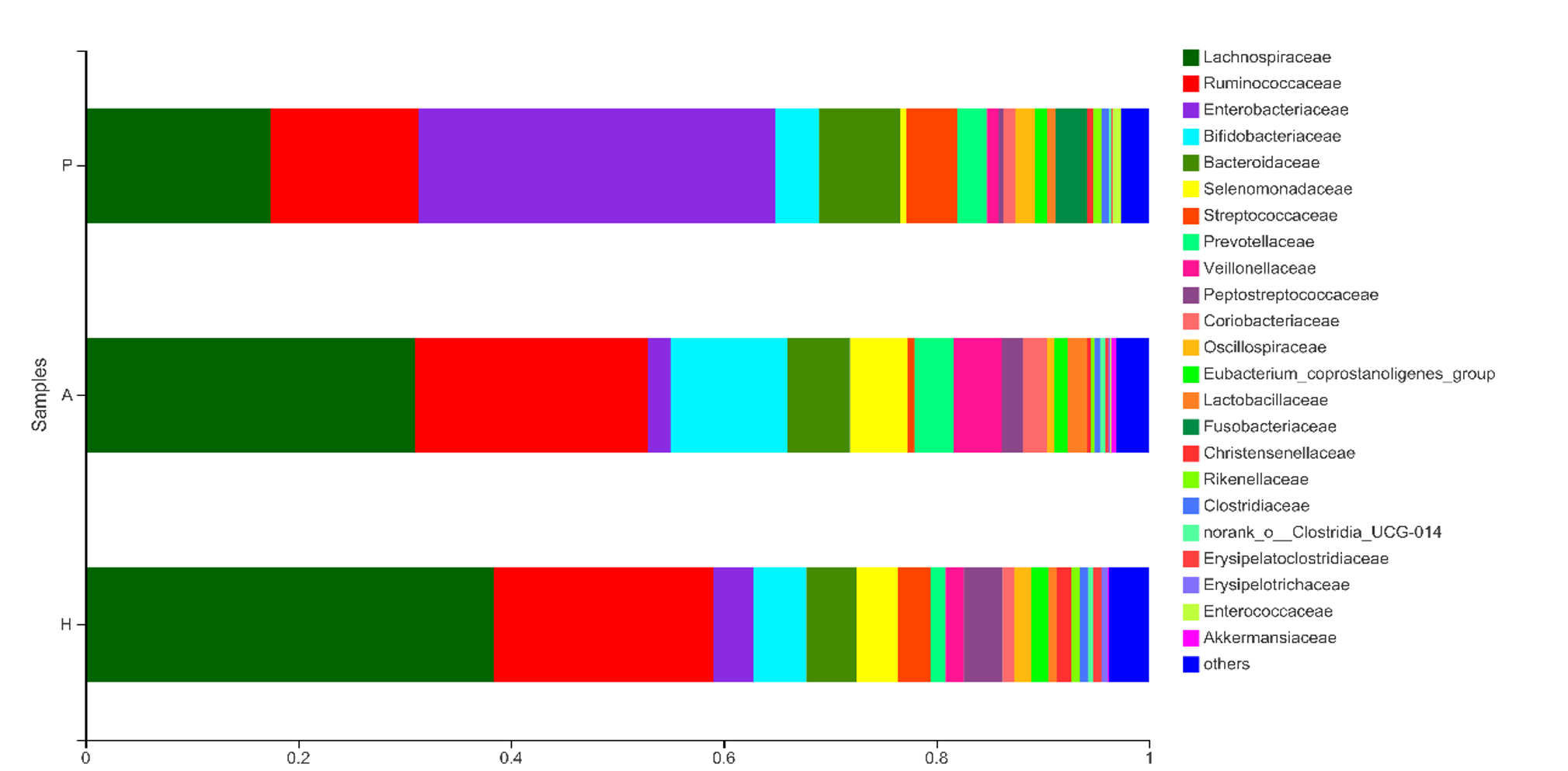

Supplement: Supplementary Figure 1 — Histograms of the different levels of bacteria. (A) Histograms on the class level. (B) Histograms on the order level. (C) Histograms on the family level. (D) Histograms on the genus level. (E) Histograms on the species level. [file Data_Sheet_1.zip › Figure S1C.TIF]

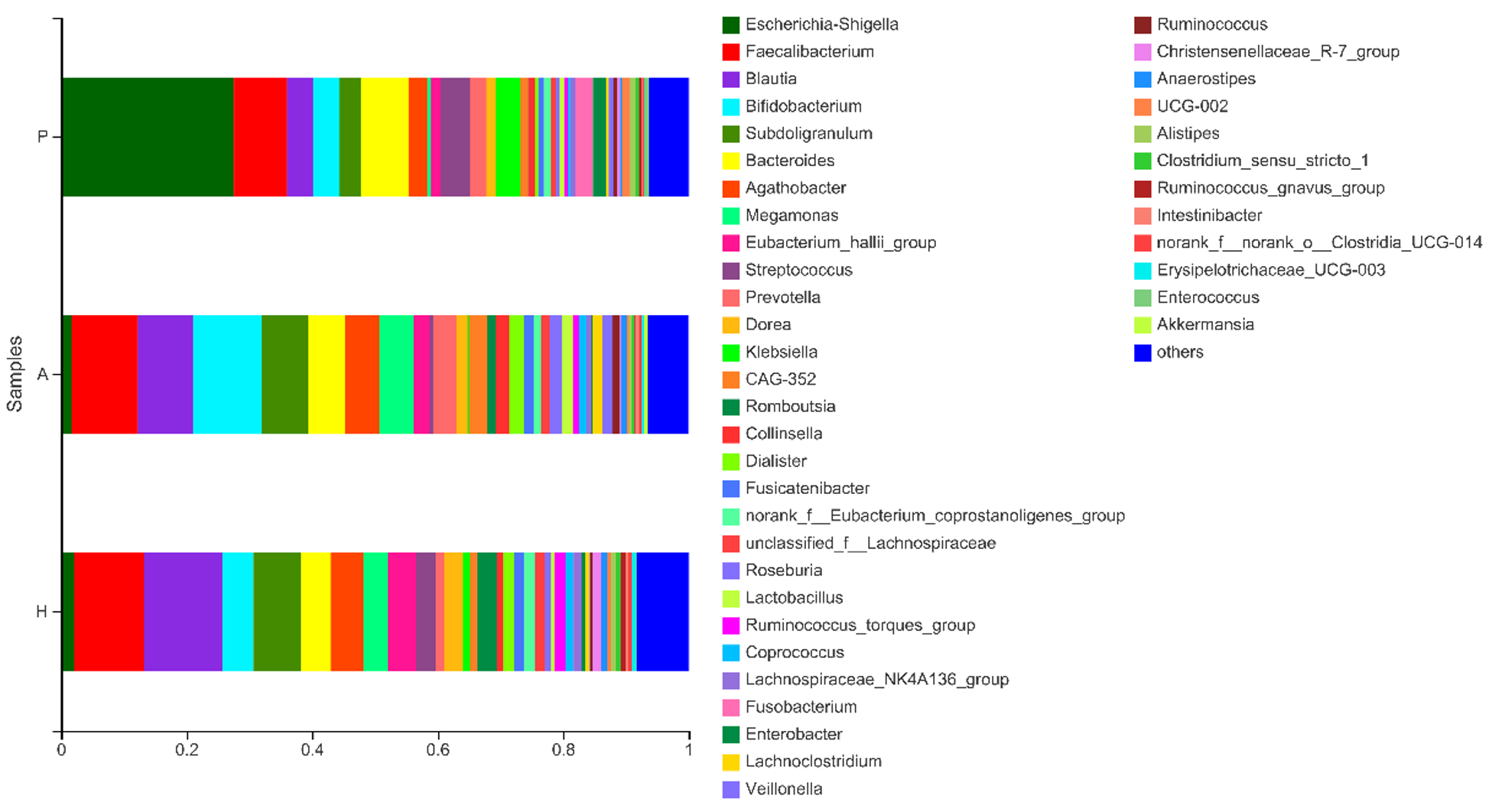

Supplement: Supplementary Figure 1 — Histograms of the different levels of bacteria. (A) Histograms on the class level. (B) Histograms on the order level. (C) Histograms on the family level. (D) Histograms on the genus level. (E) Histograms on the species level. [file Data_Sheet_1.zip › Figure S1D.TIF]

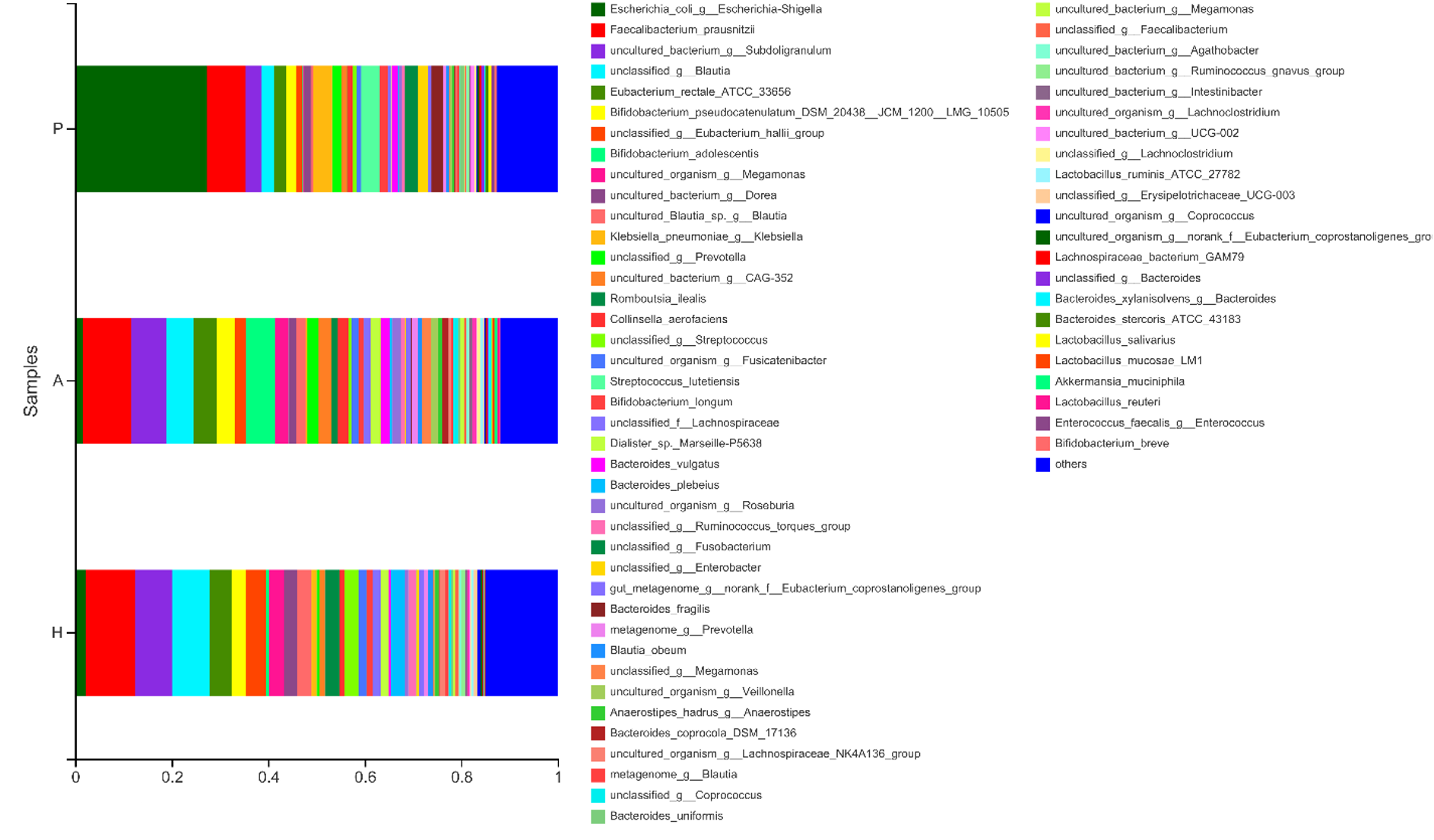

Supplement: Supplementary Figure 1 — Histograms of the different levels of bacteria. (A) Histograms on the class level. (B) Histograms on the order level. (C) Histograms on the family level. (D) Histograms on the genus level. (E) Histograms on the species level. [file Data_Sheet_1.zip › Figure S1E.TIF]

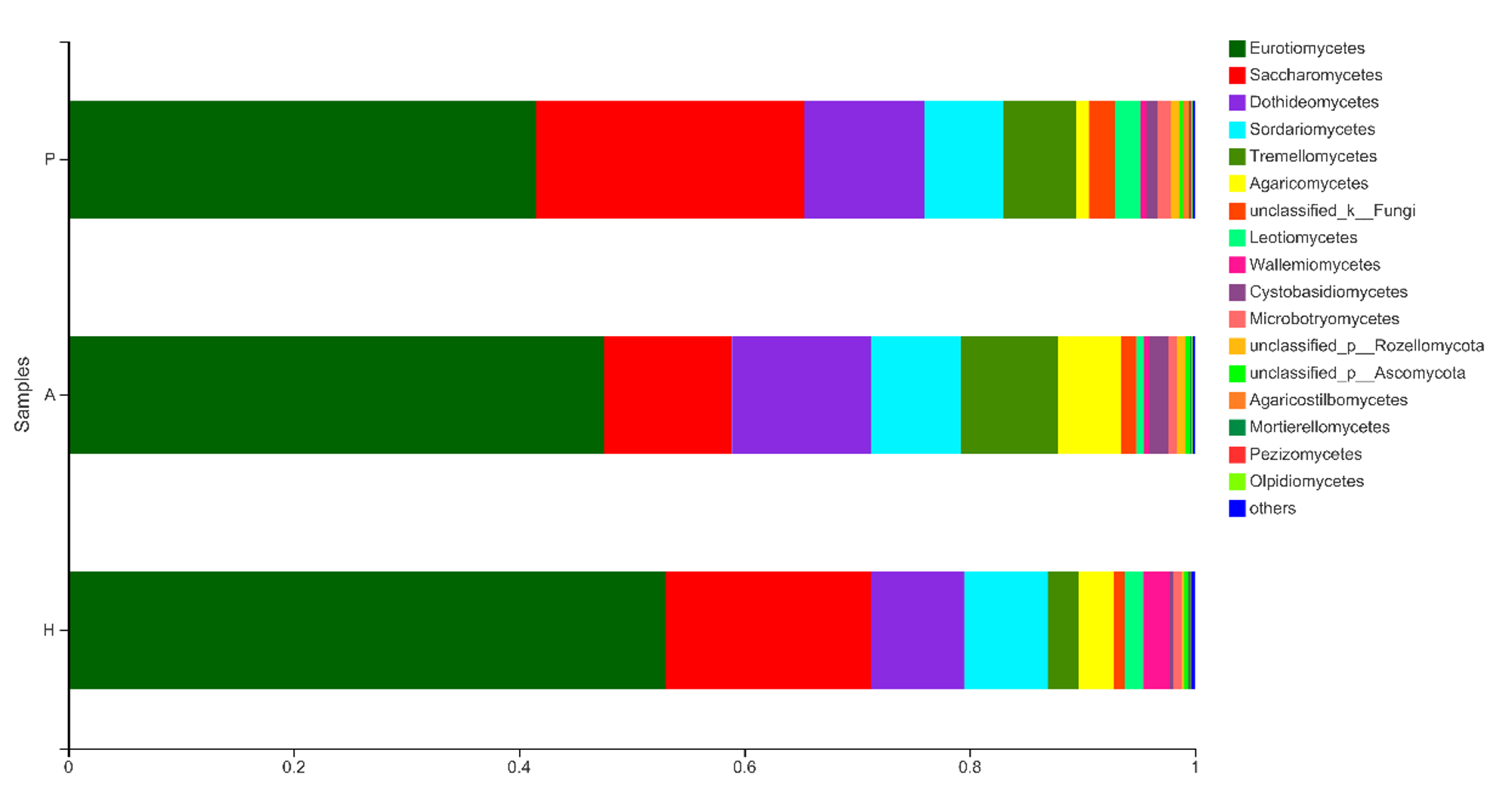

Supplement: Supplementary Figure 1 — Histograms of the different levels of bacteria. (A) Histograms on the class level. (B) Histograms on the order level. (C) Histograms on the family level. (D) Histograms on the genus level. (E) Histograms on the species level. [file Data_Sheet_1.zip › Figure S2A.TIF]

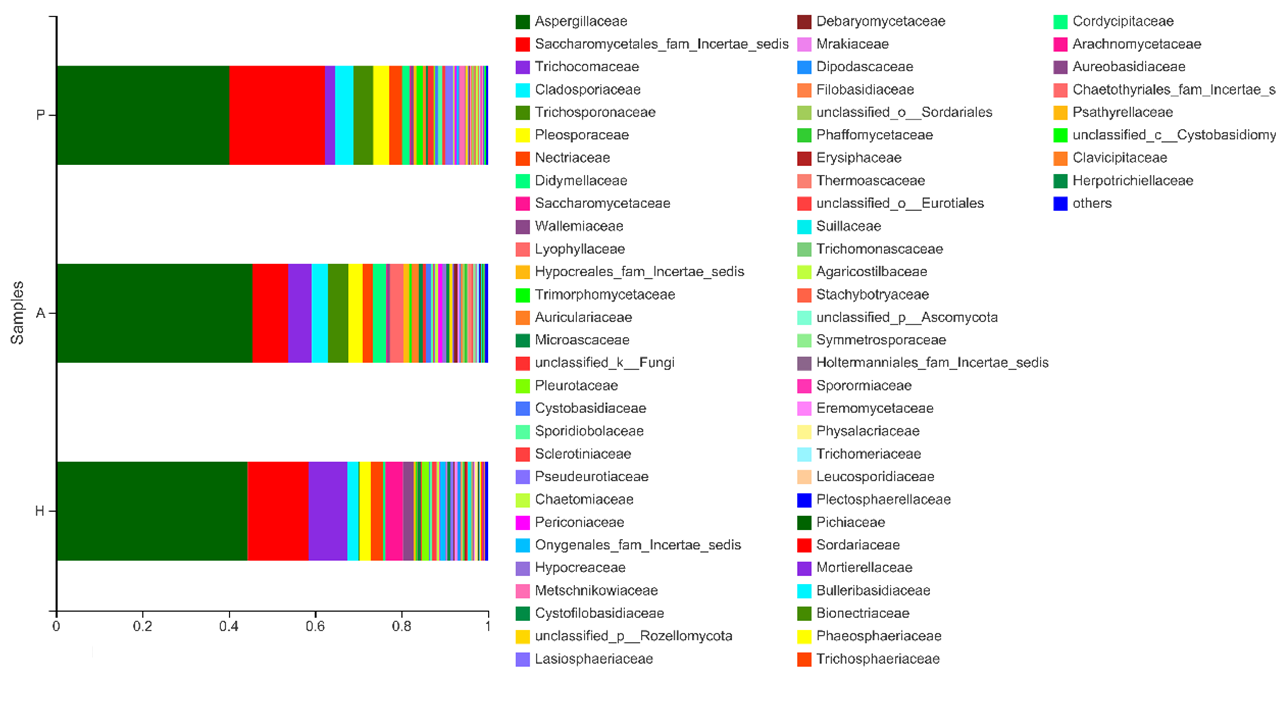

Supplement: Supplementary Figure 1 — Histograms of the different levels of bacteria. (A) Histograms on the class level. (B) Histograms on the order level. (C) Histograms on the family level. (D) Histograms on the genus level. (E) Histograms on the species level. [file Data_Sheet_1.zip › Figure S2B.TIF]

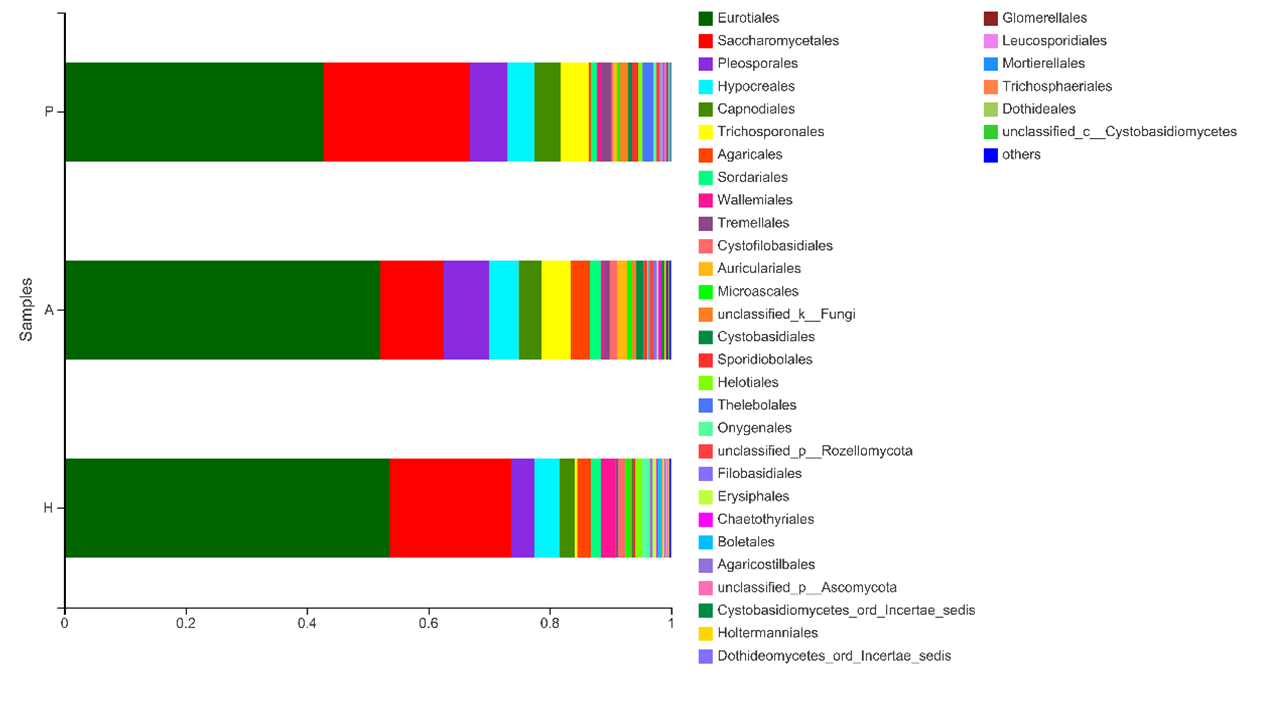

Supplement: Supplementary Figure 1 — Histograms of the different levels of bacteria. (A) Histograms on the class level. (B) Histograms on the order level. (C) Histograms on the family level. (D) Histograms on the genus level. (E) Histograms on the species level. [file Data_Sheet_1.zip › Figure S2C.TIF]

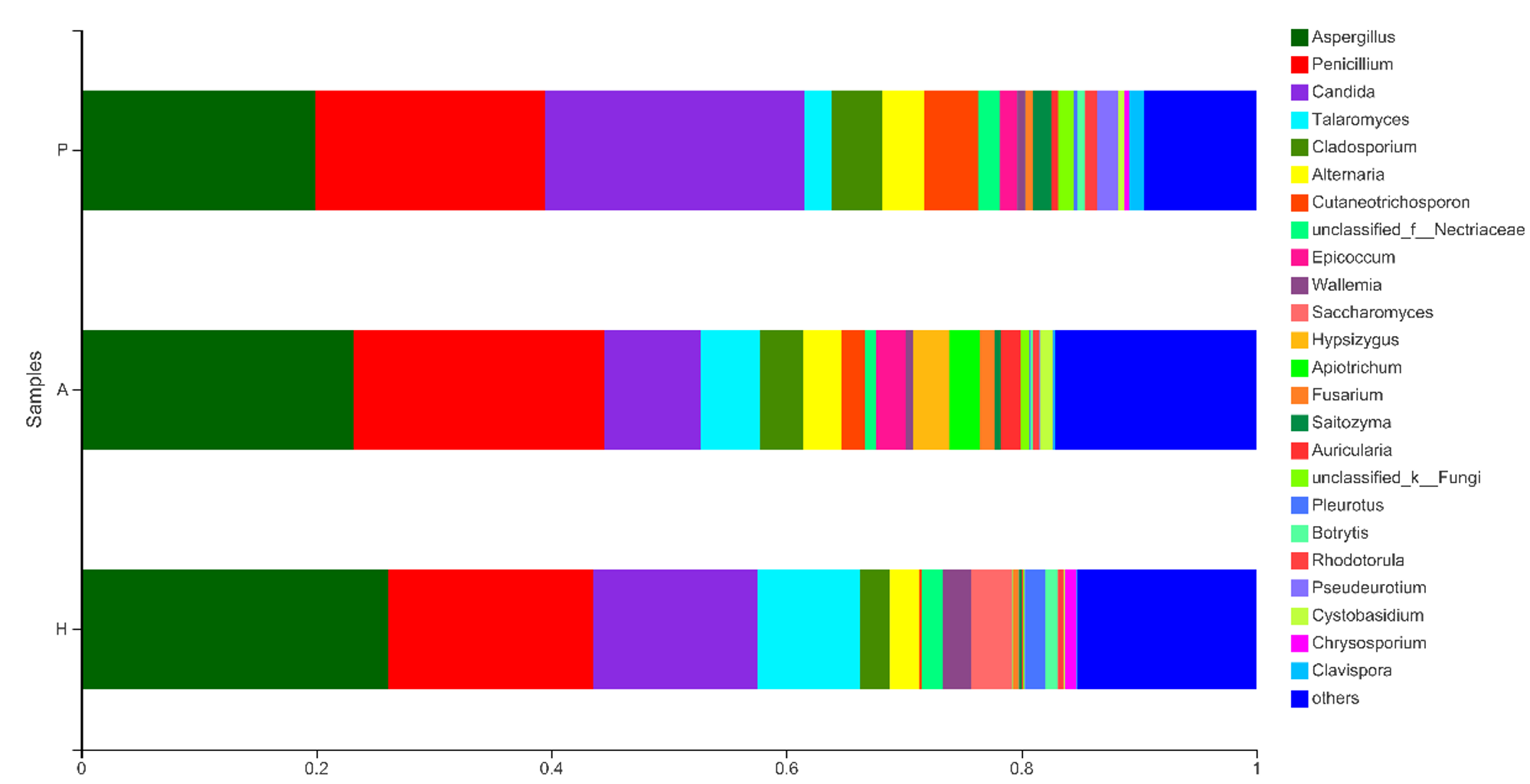

Supplement: Supplementary Figure 1 — Histograms of the different levels of bacteria. (A) Histograms on the class level. (B) Histograms on the order level. (C) Histograms on the family level. (D) Histograms on the genus level. (E) Histograms on the species level. [file Data_Sheet_1.zip › Figure S2D.TIF]

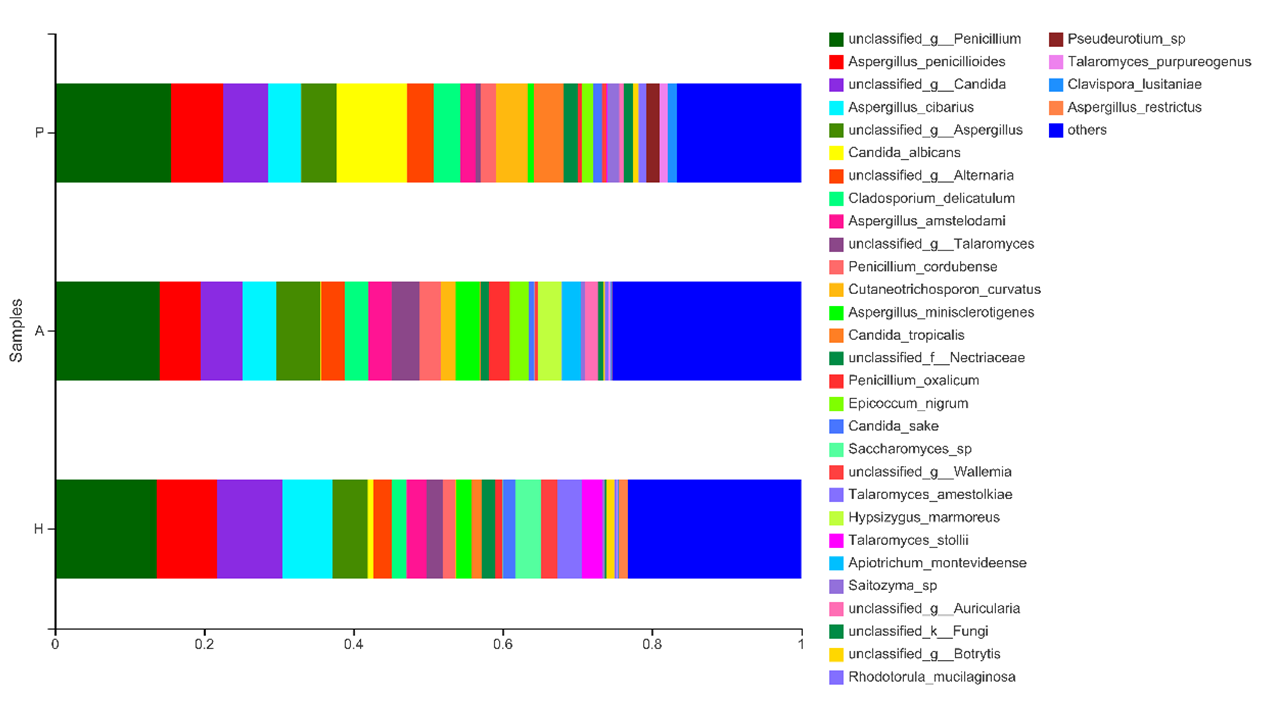

Supplement: Supplementary Figure 1 — Histograms of the different levels of bacteria. (A) Histograms on the class level. (B) Histograms on the order level. (C) Histograms on the family level. (D) Histograms on the genus level. (E) Histograms on the species level. [file Data_Sheet_1.zip › Figure S2E.TIF]

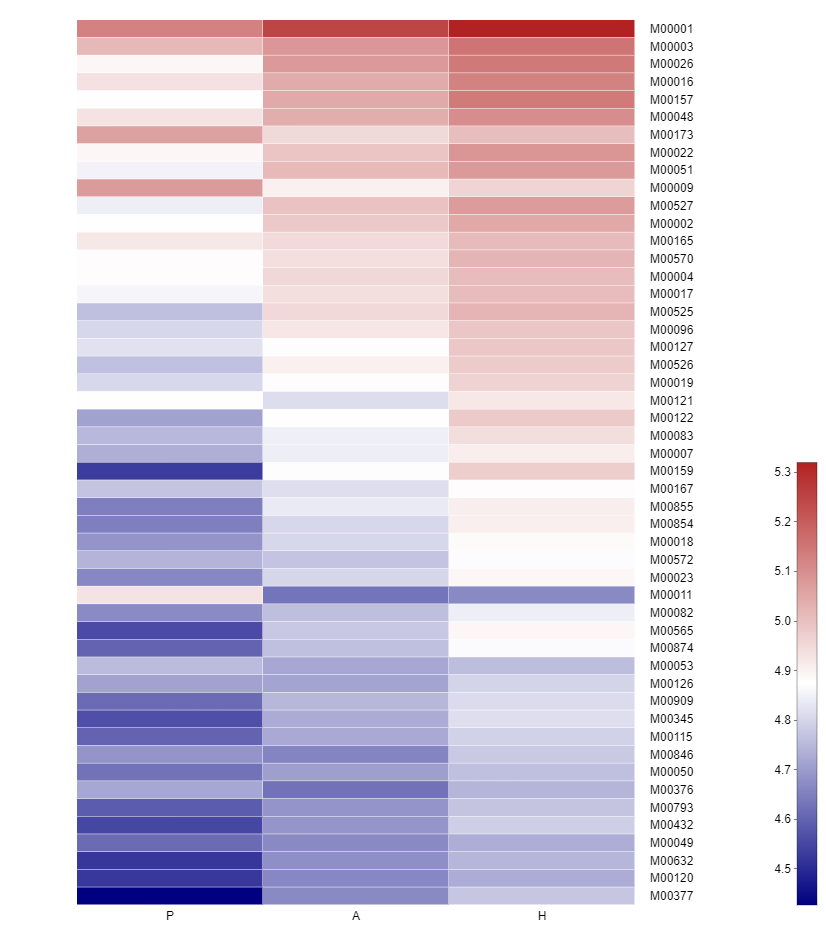

Supplement: Supplementary Figure 1 — Histograms of the different levels of bacteria. (A) Histograms on the class level. (B) Histograms on the order level. (C) Histograms on the family level. (D) Histograms on the genus level. (E) Histograms on the species level. [file Data_Sheet_1.zip › Figure S3.TIF]

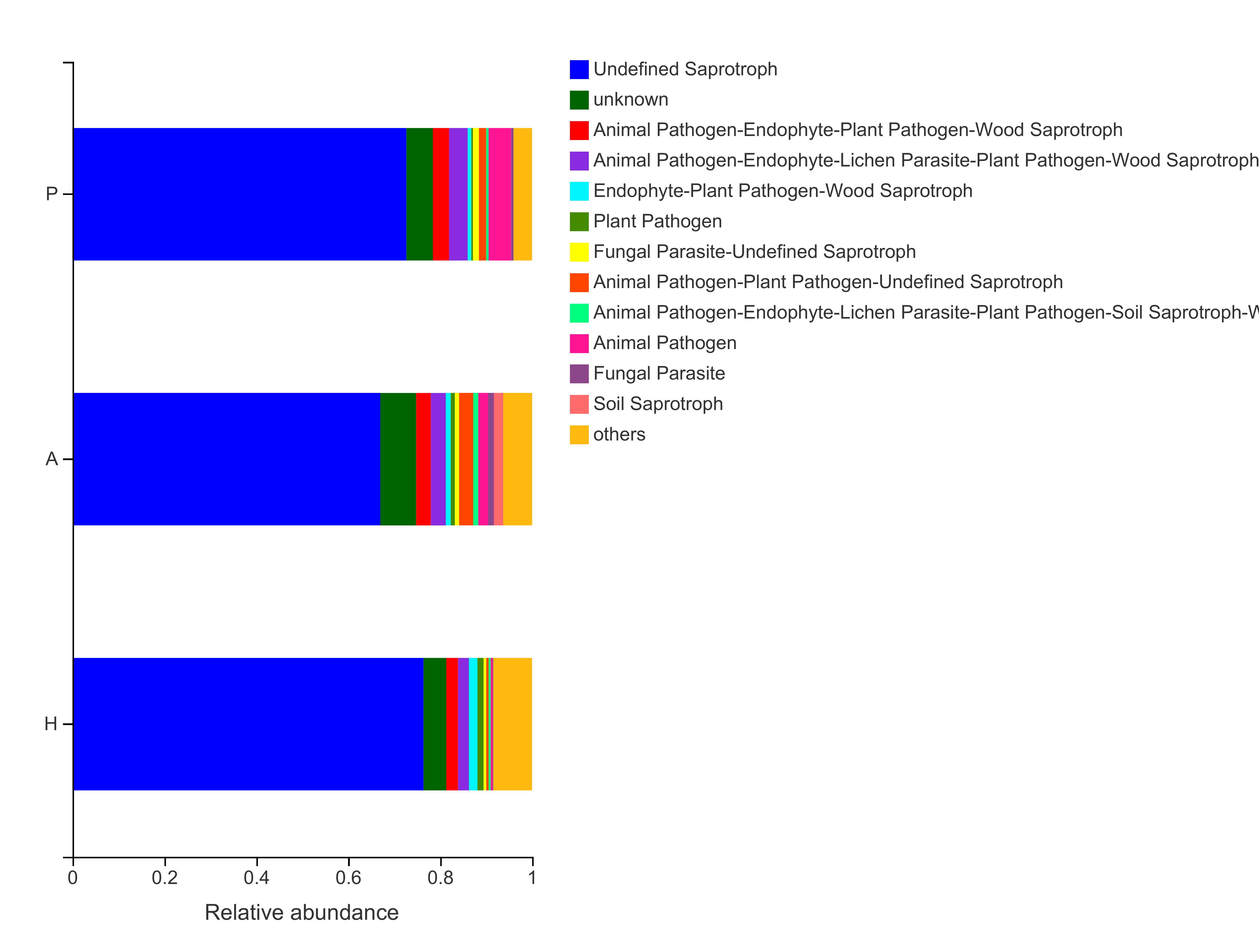

Supplement: Supplementary Figure 1 — Histograms of the different levels of bacteria. (A) Histograms on the class level. (B) Histograms on the order level. (C) Histograms on the family level. (D) Histograms on the genus level. (E) Histograms on the species level. [file Data_Sheet_1.zip › Figure S4.TIF]
